# Accelerated cerebromicrovascular senescence contributes to cognitive decline in a mouse model of paclitaxel (Taxol)‐induced chemobrain

**DOI:** 10.1111/acel.13832

**Published:** 2023-05-26

**Authors:** Chetan Ahire, Adam Nyul‐Toth, Jordan DelFavero, Rafal Gulej, Janet A. Faakye, Stefano Tarantini, Tamas Kiss, Anna Kuan‐Celarier, Priya Balasubramanian, Anna Ungvari, Amber Tarantini, Raghavendra Nagaraja, Feng Yan, Qinggong Tang, Peter Mukli, Tamas Csipo, Andriy Yabluchanskiy, Judith Campisi, Zoltan Ungvari, Anna Csiszar

**Affiliations:** ^1^ Vascular Cognitive Impairment, Neurodegeneration and Healthy Brain Aging Program, Department of Neurosurgery University of Oklahoma Health Sciences Center Oklahoma City Oklahoma USA; ^2^ Oklahoma Center for Geroscience and Healthy Brain Aging University of Oklahoma Health Sciences Center Oklahoma City Oklahoma USA; ^3^ International Training Program in Geroscience, Doctoral School of Basic and Translational Medicine/Departments of Public Health and Translational Medicine Semmelweis University Budapest Hungary; ^4^ International Training Program in Geroscience, Institute of Biophysics, Biological Research Centre ELKH Szeged Hungary; ^5^ Department of Health Promotion Sciences, College of Public Health University of Oklahoma Health Sciences Center Oklahoma City Oklahoma USA; ^6^ The Peggy and Charles Stephenson Cancer Center University of Oklahoma Health Sciences Center Oklahoma City Oklahoma USA; ^7^ International Training Program in Geroscience, First Department of Pediatrics Semmelweis University Budapest Hungary; ^8^ Stephenson School of Biomedical Engineering, Gallogly College of Engineering The University of Oklahoma Norman Oklahoma USA; ^9^ Buck Institute for Research on Aging Novato California USA

**Keywords:** aging, chemotherapy, chemotherapy‐induced cognitive impairment, dementia, functional hyperemia, senescence, vascular cognitive impairment

## Abstract

Chemotherapy‐induced cognitive impairment (“chemobrain”) is a frequent side‐effect in cancer survivors treated with paclitaxel (PTX). The mechanisms responsible for PTX‐induced cognitive impairment remain obscure, and there are no effective treatments or prevention strategies. Here, we test the hypothesis that PTX induces endothelial senescence, which impairs microvascular function and contributes to the genesis of cognitive decline. We treated transgenic p16‐3MR mice, which allows the detection and selective elimination of senescent cells, with PTX (5 mg/kg/day, 2 cycles; 5 days/cycle). PTX‐treated and control mice were tested for spatial memory performance, neurovascular coupling (NVC) responses (whisker‐stimulation‐induced increases in cerebral blood flow), microvascular density, blood–brain barrier (BBB) permeability and the presence of senescent endothelial cells (by flow cytometry and single‐cell transcriptomics) at 6 months post‐treatment. PTX induced senescence in endothelial cells, which associated with microvascular rarefaction, NVC dysfunction, BBB disruption, neuroinflammation, and impaired performance on cognitive tasks. To establish a causal relationship between PTX‐induced senescence and impaired microvascular functions, senescent cells were depleted from PTX‐treated animals (at 3 months post‐treatment) by genetic (ganciclovir) or pharmacological (treatment with the senolytic drug ABT263/Navitoclax) means. In PTX treated mice, both treatments effectively eliminated senescent endothelial cells, rescued endothelium‐mediated NVC responses and BBB integrity, increased capillarization and improved cognitive performance. Our findings suggest that senolytic treatments can be a promising strategy for preventing chemotherapy‐induced cognitive impairment.

## Graphical abstract

Graphical representation depicting the proposed mechanism of chemotherapy‐induced cognitive impairment. PTX treatment induces endothelial senescence, which impairs microvascular function and contributes to cognitive decline. PTX‐treated mice exhibit microvascular rarefaction, neuroinflammation, and impaired cognitive performance. Senescent cells are eliminated from PTX‐treated animals using genetic or pharmacological means, which results in the restoration of endothelium‐mediated neurovascular coupling responses and blood‐brain barrier (BBB) integrity, increased capillarization, and improved cognitive performance.

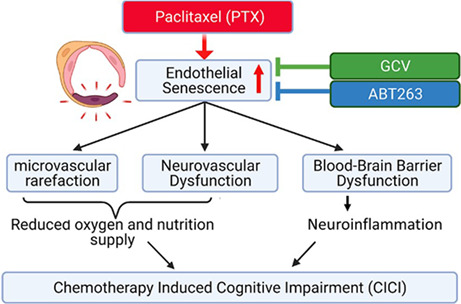

**Abbreviations**
ABT263: navitoclax
BBB: blood‐brain barrier
CICI: chemotherapy‐induced cognitive impairment
CMVECs: cerebromicrovascular endothelial cells
GCV: ganciclovir
LNAME: N(gamma)‐nitro‐L‐arginine methyl ester
NO: nitric oxide
NVC: neurovascular coupling
OCT: optical coherence tomography
PTX: paclitaxel
VSMCs: vascular smooth muscle cells

## 1 INTRODUCTION

Approximately 30% to 50% of cancer survivors experience progressive chemotherapy‐induced cognitive impairment (CICI, often referred to as “chemobrain”) (Wefel et al., 2010). Paclitaxel (PTX; sold as the brand name Taxol among others) is a taxane‐based chemotherapy drug targeting tubulin, which is used as a first‐line treatment against solid tumors including breast cancer, ovarian cancer, and cervical cancer. Although PTX has poor blood–brain barrier (BBB) penetration (Gallo et al., 2003; Kemper et al., 2003), CICI frequently occurs in patients who received PTX treatment. Most commonly affected functions include memory, attention and executive control. These symptoms persist for months or years following treatment and significantly limit the patients' quality of life and negatively affect treatment outcome. Preclinical studies show that PTX treatment also leads to a progressive impairment of cognitive function in rodent models, mimicking the clinical condition of patients with chemobrain (Chang et al., 2020). At present, there are no effective strategies to prevent or reverse PTX‐induced cognitive decline.

A paradigm shift in the pathogenesis of cognitive decline focuses on the complex role of cerebromicrovasculature. Recently, a new concept has emerged, suggesting that cancer treatments (e.g., radiation therapy) (Yabluchanskiy et al., 2020) cause microvascular injury/dysfunction, which importantly contribute to their side effects. PTX has been shown to adversely affect the phenotype of cultured endothelial cell, inhibiting proliferation and angiogenic capacity (Wang et al., 2003). Yet, the role of cerebromicrovascular alterations in PTX‐induced cognitive decline has not yet been studied.

A critical mechanism by which chemotherapeutics impair cellular and tissue function is the generation of persistent DNA damage (Demaria et al., 2017). In dividing cells, including endothelial cells, DNA damage induces a chronic stress response termed cellular senescence, which result in complex functional and phenotypic alterations (Demaria et al., 2017; Katzir et al., 2021; Kim et al., 2019; Lewis‐McDougall et al., 2019; Ogrodnik et al., 2019, 2021; Patil et al., 2019; Tuttle et al., 2020; Yousefzadeh et al., 2020). There is evidence that while mature neurons are resistant to chemotherapeutic agents, endothelial cells are highly sensitive and most chemotherapeutics cause significant molecular stresses in them. While the brain concentration of PTX is very low, endothelial cells are exposed to the highest concentrations of PTX in the body making them uniquely vulnerable to PTX‐induced DNA damage, senescence and loss of function.

**FIGURE 1:**
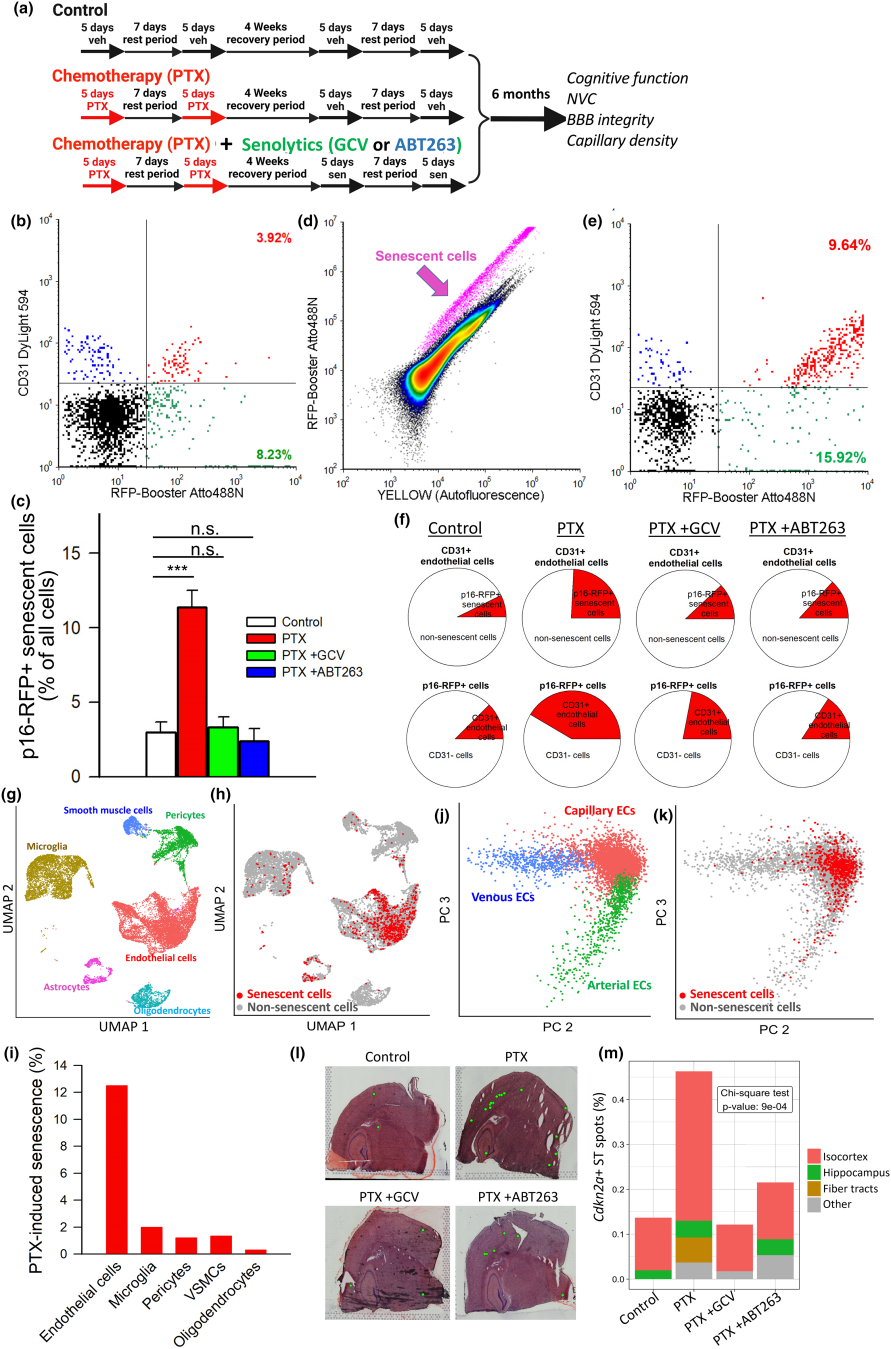
GCV and ABT263 treatment successfully deplete senescent endothelial cells from the brains of PTX‐treated p16‐3MR mice. (a) Schematic representation of the experimental design. PTX: paclitaxel, GCV: ganciclovir, NVC: neurovascular coupling. (b–f) Flow cytometric detection of RFP^+^/CD‐31^+^ senescent endothelial cells, RFP^−^/CD‐31^+^ non‐senescent endothelial cells and RFP^+^/CD‐31^−^ senescent non‐endothelial cells in single‐cell suspensions obtained from the brains of control and PTX treated p16‐3MR mice that received vehicle, GCV or the senolytic drug ABT263. Brains were analyzed 6 months after PTX. (b) shows a representative dot plot of RFP‐Booster Atto647N fluorescence (which correlates with p16‐3MR expression) versus CD‐31 staining (583/26 nm), depicting the percentage of senescent endothelial cells with bright fluorescence in the brain of a PTX treated mouse. Summary data for all senescent cells are shown in (e). Data are mean ± SEM (*n* = 4 for each data point). ***p < 0.001 versus control. (d) Representative density plots of RFP‐Booster Atto647N fluorescence versus autofluorescence obtained from the FACS sorting of single cell brain suspension of a PTX treated mouse. Note the distinct population of RFP^+^ senescent cells (purple arrow). (e) Flow cytometric detection of RFP^+^/CD‐31^+^ senescent endothelial cells and RFP^+^/CD‐31^−^ senescent non‐endothelial cells in single‐cell suspension obtained from the brain of a PTX treated p16‐3MR mouse, which was enriched for RFP+ cells by FACS. Dot plot of RFP‐Booster Atto647N fluorescence versus CD‐31 staining depicts the percentages of senescent endothelial cells with bright fluorescence in both channels and that of RFP^+^/CD‐31^−^ senescent non‐endothelial cells. (f) Pie charts in the upper row show the ratio of senescent endothelial cells as percentage of all endothelial cells. Note that PTX treatment significantly increases the presence of senescent endothelial cells in the mouse brain, which is reversed by both GCV and ABT263 treatment. Pie charts in the bottom row show the ratio of senescent endothelial cells as percentage of all senescent cells. (g) Identification of cerebromicrovascular endothelial cells based on differentially expressed marker genes (scRNAseq data). Shown is two‐dimensional UMAP plot based on differentially expressed marker genes, colored by cluster. Cluster identity was assigned based on previously reported differentially expressed genes listed in Table S1. (h) Cells with high expression of senescence markers overlaid on UMAP plots for brains of PTX treated mice. (i) Bar charts showing percentage of senescent endothelial cells, microglia, pericytes, vascular smooth muscle cells (VSMCs) and oligodendrocytes in brains of PTX treated mice. (j) Principal component analysis of RNA‐Seq data generated from brain endothelial cells derived from PTX treated mice. Capillary‐, venous‐, and arterial endothelial cells were identified based on their specific gene expression signatures. Shown is visualization of endothelial cell subclusters, color coded for the identified endothelial phenotypes. Marker genes of each cluster are provided in Table S3. Senescent endothelial cells identified based on high expression of senescence markers are highlighted (red) in (k). (l) Spatially‐resolved mRNA expression of the senescence marker gene *Cdkn2a* (green ST spots) in brains of control and PTX treated p16‐3MR mice that received vehicle, GCV or ABT263. Representative H&E stained coronal sections of brains are shown. (m) Spatial distribution of *Cdkn2a* positive spots, expressed as a percentage of all spots in the different anatomical regions in brains of each group of mice. GCV and ABT263 treatment successfully depleted senescent cells from the isocortex of PTX treated p16‐3MR mice.

Here, we test the hypothesis that PTX induces endothelial senescence *in vivo*, which contributes to cerebromicrovascular dysfunction, and thereby the genesis of cognitive decline. To achieve this goal, we used transgenic p16‐3MR mice, which allows the detection and selective elimination of senescent cells (Yabluchanskiy et al., 2020). To differentiate between cognitive symptoms caused by chemotherapy and the general comorbidity of cancer, we treated cancer‐free p16‐3MR mice with PTX or vehicle. Then, PTX‐treated and control mice were tested for cognitive performance, endothelium‐dependent neurovascular coupling (NVC) responses, brain capillary density, blood–brain barrier (BBB) integrity, and the presence of senescent cells 6 months after chemotherapy. Impaired NVC, capillary rarefaction, BBB disruption, and consequential neuroinflammation have been causally linked to the genesis of cognitive impairment (Toth et al., 2017). To establish a causal relationship between chemotherapy‐induced senescence and cerebromicrovascular dysfunction, senescent cells were depleted from two sub‐groups of PTX‐treated animals 3 months after chemotherapy using the p16‐3MR transgene or a pharmacological agent known to target senescent cells (Figure 1) (Yabluchanskiy et al., 2020).

## 2 MATERIALS AND METHODS

A detailed description of Materials and methods is available in Supplementary information.

### 2.1 Experimental animals and experimental design

To identify and eliminate senescent cells, we used a novel transgenic mouse model (p16‐3MR mice; Yabluchanskiy et al., 2020) that carries a fusion protein (3MR) under control of the p16^*Ink4a*^ promoter. 3MR contains monomeric red fluorescent protein (mRFP), which enables us to FACS‐sort senescent cells from tissues, and the herpes simplex virus thymidine kinase, which allows us to selectively kill p16‐positive senescent cells by administering the prodrug ganciclovir (GCV).

Three‐month‐old male p16‐3MR mice received PTX (5 mg/kg/day, i.p., *n* = 150) or vehicle (DMSO + saline, *n* = 50) in 2 cycles (5 days/cycle) with a 7‐day interval between cycles. Mice were left to recover for 2 weeks in the original environment. Then, PTX treated mice were assigned randomly to three groups. Two groups received the senolytic drug ABT263 (Navitoclax, Chemgood, C‐1009, i.p., 1.5 mg/kg/daily in DMSO/saline) (Yabluchanskiy et al., 2020) or ganciclovir (GCV [TSZCHEM, RG001, >99%]; i.p. 25 mg/kg/daily) for 5 days and 2 cycles with a 2 week interval between cycles (Yabluchanskiy et al., 2020). The third group served as vehicle controls.

At the end of 6 months after the PTX protocol, mice were tested for cognitive functions, NVC responses, and microvascular densityand BBB integrity, and then were euthanized for tissue collection. All animal protocols were approved by the Institutional Animal Care and Use Committee of the OUHSC.

### 2.2 Radial arms water maze testing

To determine how senescence induced by PTX and depletion of senescent cells affect cognitive function, spatial memory and long term memory were tested by assessing performance in the radial arms water maze at 6 months post‐chemotherapy, following our published protocols (Yabluchanskiy et al., 2020).

### 2.3 Spatial memory testing of mice in Y‐maze

Hippocampal‐dependent contextual memory was tested with the Y‐maze two‐trial delayed alternation task according to our published protocol (Csiszar et al., 2013).

### 2.4 Grip strength, rotarod, and gait analysis

As an additional control, we determined how the pharmacological treatments affect muscle function in mice. To that end, the grip strength test was used to measure the maximal muscle strength of mouse forelimbs. Motor coordination was assessed using an automated four‐lane rotarod tool. To determine the impact of PTX treatment on gait coordination, we tested the experimental groups of mice using an automated computer assisted method (CatWalk; Noldus Information Technology Inc.) using our previously reported protocol (Ungvari et al., 2017).

### 2.5 Intravital two‐photon microscopy

To assess BBB permeability and cerebromicrovascular density, mice were equipped with a chronic cranial window and intravital two‐photon microscopy‐based and optical coherence tomography (OCT) based imaging methods were used as previously described (Nyul‐Toth et al., 2021).

### 2.6 Assessment of neurovascular coupling responses

NVC responses were assessed as described previously (Tarantini, Balasubramanian, et al., 2021; Tarantini et al., 2018, 2021). In brief, mice in each group were anesthetized with isoflurane (2% induction and 1% maintenance), endotracheally intubated, and ventilated. The skull was thinned and changes in cerebral blood flow (CBF) were assessed above the left somatosensory whisker barrel cortex in response to mechanical stimulation of the right whiskers. To assess the role of NO mediation, CBF responses to whisker stimulation were repeated after administrating the nitric oxide synthase inhibitor N^ω^‐Nitro‐L‐arginine methyl ester (L‐NAME). At the end of the experiments, the animals were transcardially perfused with ice‐cold PBS and decapitated. The brains were immediately removed and samples were collected for subsequent studies. Whole brains were collected for FACS analysis and transcriptomics. Acute brain slices were collected for LTP measurements. Half brains were immersion fixed in 4% paraformaldehyde for 24 hours, transferred to sucrose gradients and embedded and cut for immunohistochemistry.

### 2.7 Electrophysiological studies to assess synaptic function and long‐term potentiation (LTP)

To determine how PTX affects neuronal function, extracellular recordings were performed from acute hippocampal slices obtained from a separate cohort of control and PTX‐treated animals as described (Tucsek et al., 2017).

### 2.8 Determination of senescent cell burden by flow cytometric analysis

We used sorted cells obtained from the single‐cell suspensions from the brain samples to analyze senescent cell burden using our published protocols (Yabluchanskiy et al., 2020). In brief, single‐cell suspensions were prepared. Fixed cells were stained with the RFP‐Booster (AlexaFluor‐488, 1:1000; Chromotek; US‐QUO201590, 0.5 gm/L) for 30 min, centrifuged (300 × g, 10 min), and resuspended in MACS buffer (Miltenyi Biotech). The RFP‐Booster allows for the detection of senescent cells that express the RFP‐containing 3MR construct under the control of the p16^*Ink4a*^ promoter.

To assess senescent cell burden, a portion of the RFP‐Booster‐stained samples was also labelled with an antibody directed against the endothelium‐specific surface marker CD31. First, the fraction of RFP+ senescent cells were determined as a percentage of total cells in the single cell suspensions from whole brain lysates using a Guava® EasyCyte™ BGR HT Flow Cytometer (Luminex). Then, the ratio of RFP^+^/CD31^+^ senescent endothelial cells as a percentage of all CD31^+^ endothelial cells were determined.

Fluorescent activated cell sorting (FACS) with the low‐pressure WOLF Cell Sorter™ (NanoCellect) was used to obtain a cell suspension enriched in brain senescent cells. RFP+ senescent cells were stained with cell‐specific markers. Antibodies against CD31 were used to quantify the ratio of senescent endothelial cells.

### 2.9 Assessing PTX‐induced senescence in cultured cerebromicrovascular endothelial cells

Cellular senescence is characterized by senescence‐associated β‐galactosidase (SA‐β‐gal) activity. To assess the sensitivity of cultured primary cerebromicrovascular endothelial cells to PTX‐induced senescence, we measured SA‐β‐gal activity in PTX‐treated cultured human cerebromicrovascular endothelial cells (CMVECs). Human CMVECs were treated with vehicle (DMSO in media) or PTX (Ramanathan et al., 2005) for 10 days. To assess the sensitivity of CMVECs to PTX‐induced senescence, SA‐β‐gal activity was compared in PTX‐treated CMVECs and untreated controls. The percentage of β‐galactosidase‐positive cells (blue cytoplasmic staining) was calculated by a naïve observer.

### 2.10 Single‐cell transcriptomics

To identify senescent cells in brains of PTX‐treated mice, a single‐cell transcriptomics‐based method was also used, as described (Kiss et al., 2020). This technology enables capture of mRNAs from single cells obtained from dissociated tissues, synthesis and amplification of cDNA, and generation of single‐cell libraries for sequencing. We used a gel bead‐in‐emulsion‐based droplet sequencing method, which is ideal for studying a large amount of brain cells in an unbiased manner. We identified cerebromicrovascular endothelial cells and other brain cell types on the basis of their gene expression profile and matched transcriptomic signatures of cellular senescence to these cells, as previously described (Kiss et al., 2020). Cell clusters were identified by the expression of known, previously validated canonical cell type markers (Kiss et al., 2020) (Table S1). The set of senescence‐related core genes used for gene set enrichment analysis is given in Table S2. For further analysis, endothelial cells were divided into three sub‐clusters: endothelial cells form arteries, veins and capillaries using canonical marker genes (Table S3).

### 2.11 Spatial transcriptomics

Spatial transcriptomics (ST) was used to assess spatial distribution of senescent cells in brains of PTX‐treated mice, as previously described (Kiss et al., 2022). Spatially resolved whole transcriptome mRNA expression was analyzed in sections of brains, while capturing histological information in the same tissue section. Microdomains containing senescent cells were identified on the basis of their senescence‐related gene expression profiles and were mapped to different anatomical brain regions, including the isocortex, white matter and hippocampi as described (Kiss et al., 2022).

### 2.12 Detection of activated microglia by immunohistochemistry

Brains were perfusion‐fixed and frozen OCT‐embedded sagittal sections (35 μm) were cut. Sections were immunolabeled for IBA‐1 (rabbit anti‐mouse Iba‐I antibody; 1:200, Fijifilm; overnight at 4°C) and the endothelial marker endomucin (rat monoclonal anti‐mouse endomucin antibody; 1:50, Invitrogen; overnight at 4°C) to identify microglia and capillary endothelial cells in the brain, respectively. Confocal images were obtained using Leica SP8 MP confocal laser scanning microscope. The relative staining intensity for IBA‐1 positive perivascular microglia per region of interest was assessed.

### 2.13 Statistical analysis

Depending on the experiment, statistical analyses were carried out by unpaired *t* test, one‐way or two‐way ANOVA with Fisher LSD *post hoc* test using GraphPad Prism 7.0, as appropriate. Differences were considered significant at *p* < 0.05. Data are presented as means ± standard error of mean (SEM) or all measured values as bar graphs or box plots with interquartile distributions and median values. Analysis was made with GraphPad Prism.

## 3 RESULTS

### 3.1 PTX induces senescence in cultured endothelial cells

To provide prima facie evidence that PTX induces senescence in endothelial cells, we performed SA‐β‐gal staining of PTX treated and untreated cerebromicrovascular endothelial cell (CMVEC) cultures. PTX significantly increased the ratio of CMVECs with positive SA‐β‐gal staining (Figure S1).

### 3.2 PTX induces endothelial senescence: Protective effects of senolytic treatments

To elucidate the effect of *in vivo* PTX treatment on cellular senescence and to determine the efficacy of senolytic treatments, we used flow cytometry to detect p16‐RFP+ senescent cells 6 months after PTX treatment (Figure 1b). PTX treatment resulted in significant increases in the number of p16‐RFP+ cells in the mouse brain (Figure 1c). Conversely, both GCV and ABT263 treatment reduced p16‐RFP+ senescent cell burden in brains of PTX treated mice (Figure 1c).

To demonstrate how PTX affects endothelial cells, samples enriched for senescent p16‐RFP+ cells (Figure 1d) were co‐sorted for the endothelial marker CD31 (Figure 1e). We found an increased fraction of CD31+/p16‐RFP+ among the p16‐RFP+ cells isolated from the brains of PTX treated mice, indicating that PTX induces significant endothelial senescence (Figure 1f). Further, flow cytometric analysis confirmed that an increased fraction of CD31+ endothelial cells express the 3MR protein post‐PTX treatment (Figure 1f).

FACS analysis showed that in PTX treated mice both GCV and ABT263 reduced the ratio of CD31+/p16‐RFP+ among the p16‐RFP+ cells (Figure 1f). Additionally, both GCV and ABT263 treatment reduced the percentage of p16‐RFP+/CD31+ senescent endothelial in brains of PTX treated mice, supporting the concept that these senolytic treatments successfully eliminated senescent endothelial cells (Figure 1f).

### 3.3 Characterization of PTX‐induced senescence by scRNA‐seq

Single‐cell transcriptomes were validated by quality control measures (Figure S2a,b). Unbiased Louvain clustering of cells resolved 6 robust, transcriptionally distinct clusters of cells (Figure 1g). Cell clusters were identified by the significant, cluster specific markers calculated by the MAST method (Table S1). Using this method endothelial cells could be clearly identified. Examples for the expression of canonical endothelial cell markers overlaid on UMAP plots of all cells are shown in Figure S2c–f. Other cell types identified included microglia, smooth muscle cells, pericytes, and oligodendrocytes and a small number of astrocytes (Figure 1g).

To identify senescent subgroups of endothelial cells and other cell types we first examined the expression of *Cdkn2a* and other commonly used senescence markers (Table S2). Because single cell sequencing is inherently prone to dropout, or incomplete detection of genes expressed at low levels, we calculated a modified enrichment score for the whole senescence marker gene set for each cell as described in the Methods. A major advantage of this method is that it considers both the expression amplitude of senescence‐related genes and the number of senescence‐related genes expressed in each cell. We found that in brains of PTX treated mice a populous subgroup of senescent endothelial cells (characterized by high senescence gene enrichment scores) is present (Figure 1h,i), whereas other cell types were less affected. Endothelial cells exhibit significant transcriptomic heterogeneity (Kalucka et al., 2020). We defined arterial, capillary, and venous endothelial cell subclusters using previously identified specific markers (Kalucka et al., 2020) (Figure 1j). PTX predominantly induced senescence in capillary endothelial cells (Figure 1j).

To assess localization of senescent cells, we used spatial transcriptomics and analyzed expression of *Cdkn2a*. Figure 1l depicts the spatially‐resolved expression of *Cdkn2a* in the brains of control and PTX treated p16‐3MR mice that received vehicle, GCV or ABT263. We found that in brains of PTX treated mice compared to control brains there is a more populous group of senescent cells, indicated by the increased ratio of *Cdkn2a* positive ST spots (Figure 1m). Analysis of the spatial distribution of *Cdkn2a* positive spots indicated that senescent cells are more prevalent in the isocortex and the white matter, as compared to brains of control mice (Figure 1m). Both GCV and ABT263 reduced the senescent cell burden in these brain regions (Figure 1m).

### 3.4 PTX promotes microvascular rarefaction: Protective effects of senolytic treatments

**FIGURE 2:**
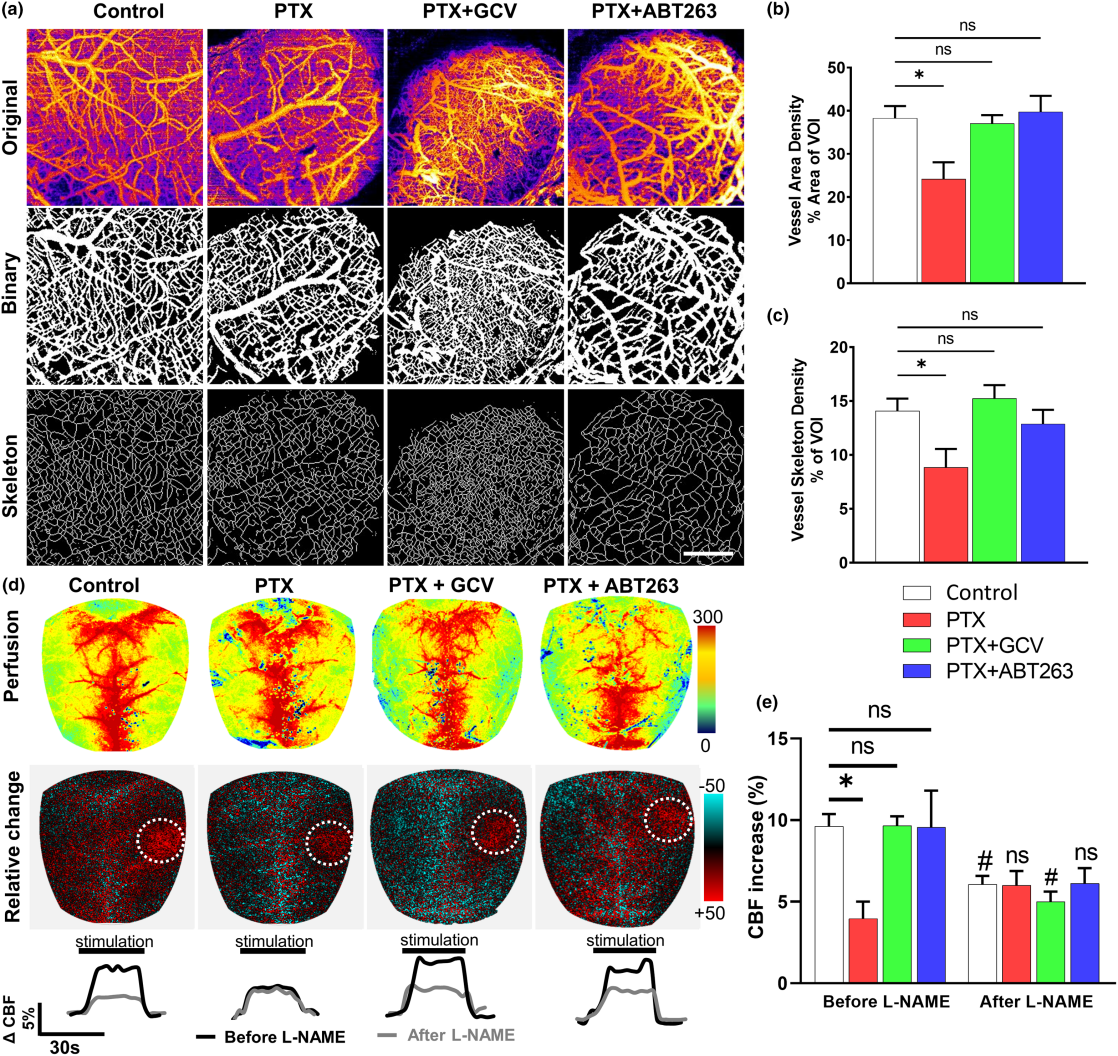
Elimination of senescent cells increases capillary density and improves endothelium‐mediated neurovascular coupling responses in PTX‐treated mice. (a) Segmentation of blood vessels in brain parenchyma on OCT images. Original z‐stack images acquired in brains of control and PTX treated p16‐3MR mice that received vehicle, ganciclovir (GCV) or ABT263 were maximum projected. Thresholded binary images and skeletons were used to calculate indices (vascular density index, vessel skeleton density index) corresponding to microvascular density. (b,c) Microvascular density was significantly decreased by PTX treatment and rescued by treatment with GCV or ABT263. Data are mean ± SEM. *p < 0.05. (d) Representative pseudocolour laser speckle flowmetry maps of baseline CBF (upper row; shown for orientation purposes) and CBF changes in the whisker barrel field relative to baseline during contralateral whisker stimulation (bottom row, right oval, 30 s, 5 Hz) in control and PTX treated p16‐3MR mice that received vehicle, GCV or ABT263. Color bar represents CBF as percent change from baseline. The NO synthase inhibitor L‐NAME was administered to test NO mediation of functional hyperemia. Bottom: time‐course of CBF changes after the start of contralateral whisker stimulation (horizontal bars). Summary data are shown in (e). Data are mean ± SEM (*n* = 8–9 in each group), *p < 0.05 versus control; ^#^ *p* < 0.05 versus before L‐NAME. (one‐way ANOVA with *post‐hoc* Tukey's tests). n.s., not significant. Capillary density and NVC responses were assessed 6 month post‐PTX treatment (see Materials and methods).

To determine the effects of PTX on the structural integrity of the cerebral microcirculation, we compared microvascular densities using OCT and two‐photon microscopy in the mouse cortex (Nyul‐Toth et al., 2021). Briefly, z‐stack two‐photon images from the WGA‐A594 channel (Figure S5) and OCT recordings (Figure 2a) went through dimension reduction by maximum intensity projection. Contrast was enhanced, noise was removed, then the images were converted into binary and pixel values were measured and visualized as skeleton images of the vascular network (Figure 2). Both vessel area density indices and vessel skeleton density indices (which are independent of the size distribution of vessels in the VOI) were compared. Using both two‐photon microscopy (Figure S5) and OCT (Figure 2b,c), we found that these vascular density indices were significantly decreased in the cortices of PTX treated mice, as compared to controls, indicating that PTX causes cerebromicrovascular rarefaction. Both GCV and ABT263 increased microvascular density in PTX treated mice (Figure 2b,c and Figure S5).

### 3.5 PTX impairs endothelial NO‐mediated NVC responses: Protective effects of senolytic treatments

To determine whether endothelial senescence impairs endothelium‐mediated NVC responses, we assessed functional hyperemia in the whisker barrel cortex in mice 6 months after PTX treatment. CBF responses in the whisker barrel cortex elicited by contralateral whisker stimulation were significantly decreased in PTX treated mice compared to control animals indicating impaired NVC (representative laser speckle contrast images and CBF tracings are shown in Figure 2d summary data are shown in Figure 2e).

Consistent with an important role for endothelial NO in mediating functional hyperemia (Balasubramanian et al., 2020; Tarantini, Nyul‐Toth et al., 2021), administration of the eNOS inhibitor L‐NAME significantly decreased NVC responses in control animals (*n* = 8–9) (representative CBF tracings are shown in Figure 2d, summary data are shown in Figure 2e). In contrast, in PTX treated animals NVC responses were unaffected by L‐NAME, indicating that PTX impairs NVC responses mediated by endothelium‐derived NO (Figure 2d). In PTX treated mice removal of senescent cells by GCV or ABT263 significantly increased CBF responses induced by contralateral whisker stimulation, restoring NVC to levels observed in control mice (Figure 2d,e). In GCV and ABT263‐treated PTX mice L‐NAME significantly decreased CBF responses elicited by whisker stimulation (Figure 2d,e), suggesting that removal of senescent cells restored mediation of NVC responses by endothelium‐derived NO.

In theory, PTX could reduce functional hyperemia by impairing neuronal activation. To examine this possibility, we measured evoked neuronal activation by assessing field excitatory postsynaptic potentials in hippocampal preparations from control and PTX treated mice. PTX treatment did not affect neuronal function and synaptic activity (Figure S6). Therefore, PTX is unlikely to contribute to impaired functional hyperemia by modulating neuronal activation.

### 3.6 PTX promotes BBB disruption and neuroinflammation: Protective effects of senolytic treatments

**FIGURE 3:**
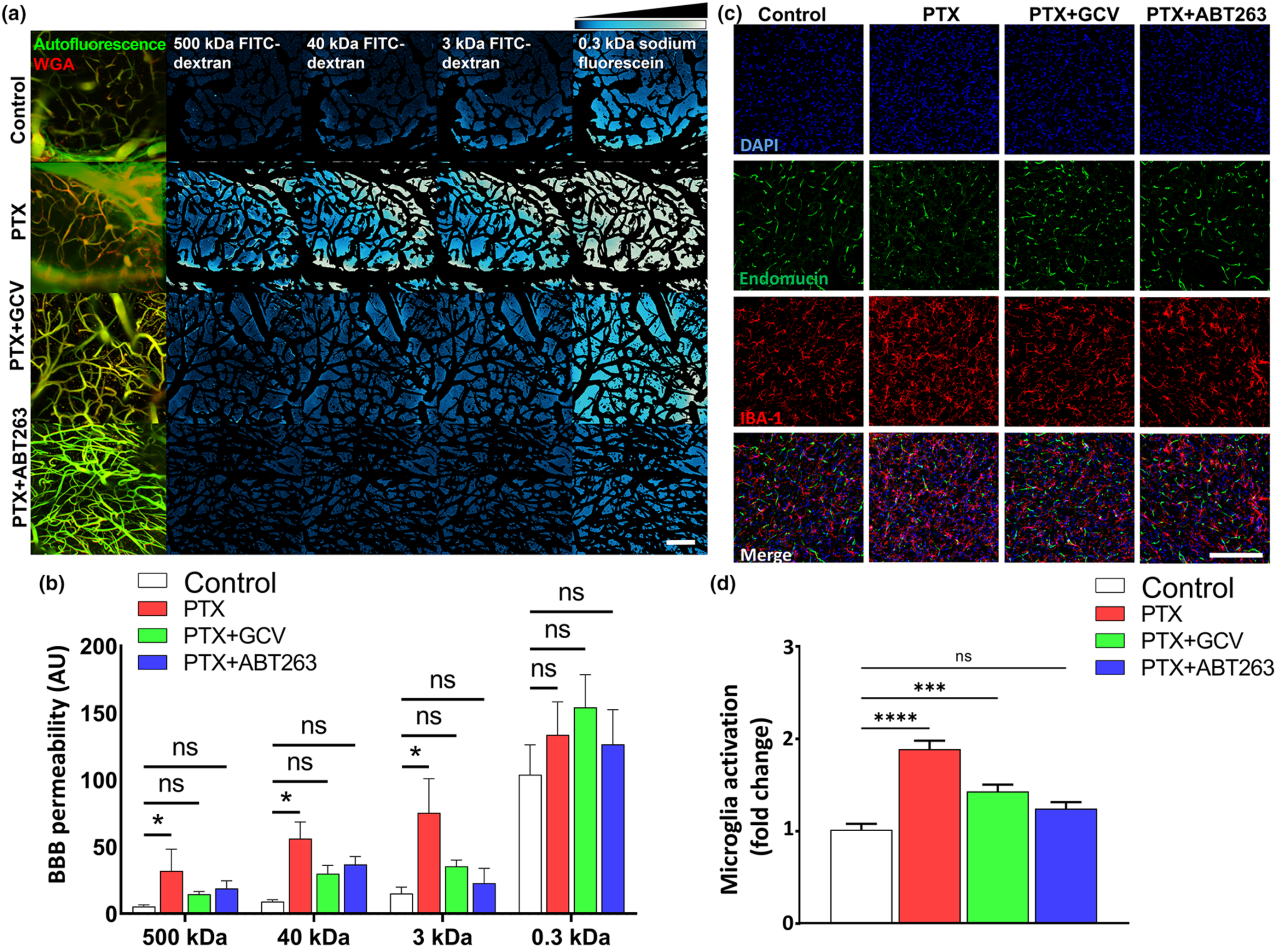
Elimination of senescent cells rescues blood brain barrier integrity and attenuates neuroinflammation in PTX‐treated mice. (a) Two‐photon‐imaging‐based measurement of microvascular permeability to fluorescent tracers in brains of control and PTX treated p16‐3MR mice that received vehicle, ganciclovir (GCV) or ABT263. Left: Z‐stack projection of cerebral vasculature. Red fluorescence: WGA‐Alexa594 staining of the glycocalyx of the endothelial cells. Green autofluoresent background in the tracer channel is shown. Right: Changes in tracer fluorescence intensity in the extravascular space and brain parenchyma in each group of animals upon injection of the fluorescent tracers of different molecular weight. Images captured subsequent to tracer administrations were maximum projected (z‐stack) and subtraction of the images of WGA‐Alexa594 stained vasculature was performed. Intensity plots derived from median projection of time‐stacks of the parenchymal recordings show increased extravasation of tracers of different molecular weights in brains of PTX‐treated mice as compared to those of control mice. Note that treatment with GCV or ABT263 attenuates BBB disruption. Relative fluorescent intensity scale is shown at top right. Scale: 100 μm. (b) Summary data for microvascular permeability to fluorescent tracers with different molecular weights in brains of each group of animals. Note the increased permeability for the different sized tracers in PTX‐treated animals and reversal by senolytics. Data are mean ± SEM. *p < 0.05. (*n* = 5–10 for each datapoint). (c) Confocal images showing perivascular IBA1 positive microglia (red fluorescent cells located adjacent to green fluorescent endomucin positive capillaries; blue fluorescence: nuclear staining with DAPI) in brains of control and PTX treated p16‐3MR mice that received vehicle, GCV or ABT263. (d) Bar graphs are summary data of relative changes in activated microglia. Data are mean ± SEM (*n* = 4 for each datapoint). ***p < 0.001.

Using two‐photon microscopy, the relative permeability of the fluorescent tracers was measured. Figure 3a shows background‐corrected fluorescent intensity changes over time in the brain parenchyma in mice from each experimental group after the administration of different size tracers. As a measure of relative vascular permeability, the area under curve of normalized intensity changes as a function of time was calculated in each group. For the 500, 40, and 3 kDa tracers the cerebral microcirculation of PTX treated animals exhibited significantly increased permeability (Figure 3b). In PTX treated mice removal of senescent cells by GCV or ABT263 significantly attenuated BBB permeability (*n* = 5–10), restoring it to levels observed in control mice (Figure 3b). This observation is consistent with the concept that endothelial senescence‐related disruption of the BBB is predominantly due to the increased transcellular permeability. Leakage of plasma‐derived factors through the damaged BBB can induce neuroinflammation by activating microglia (Tucsek et al., 2014). We found that the number of IBA1+ microglia was increased in the hippocampi of PTX‐treated mice, whereas it was normalized to control levels by removal of senescent cells via GCV or ABT263 treatment (Figure 3c,d).

### 3.7 PTX impairs cognitive performance in mice: Protective effects of senolytic treatments

**FIGURE 4:**
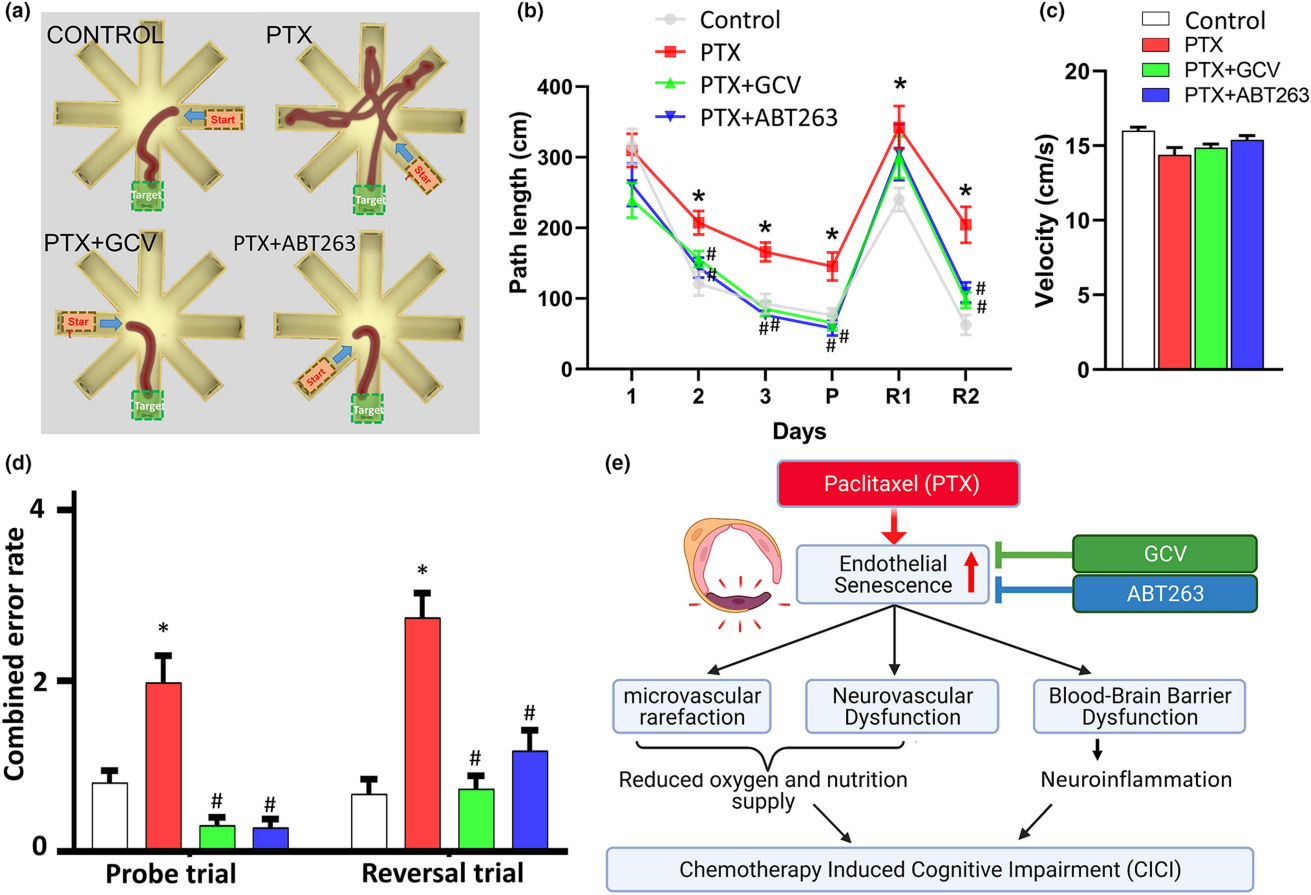
Rescue of microvascular function by elimination of senescent cells improves performance of PTX treated p16‐3MR mice in the radial‐arm water maze (RAWM). Control p16‐3MR mice and PTX treated p16‐3MR mice that received vehicle, ganciclovir (GCV) or ABT263 were tested in the RAWM. (a) Representative probe test search path of a randomly selected animal from each group is shown with the target position highlighted in green. Note the PTX treated mice that received vehicle required more time and a longer path length in order to find the hidden escape platform than both control animals and GCV‐ or ABT263 treated PTX mice. The PTX treated mouse also entered multiple incorrect arms. (b) PTX treated animals have longer path lengths throughout day 2 and 3 of the learning phase, and the retrieval day 10 (“P”) as compared to control animals. Both treatment with GCV and ABT263 improved learning and memory performance. (c) PTX treated animals also made significantly more errors during probe trial and probe reversal than control animals. In contrast, PTX treated mice treated with GCV or ABT263 perform this task significantly better than vehicle treated PTX mice. Data are mean ± SEM (*n* = 10–15 for each data point). *p < 0.05 versus control, ^#^ *p* < 0.05 versus vehicle treated PTX. (d) Scheme depicting the microvascular mechanisms (microvascular rarefaction, neurovascular dysfunction and BBB disruption) by which PTX‐induced endothelial senescence may contribute to the genesis of chemotherapy‐induced cognitive impairment.

To test whether mice exhibit significant impairment of hippocampal encoded functions of learning and memory after chemotherapy and to determine the underlying mechanisms, we assessed the effects of senolytic treatments on learning and memory function in mice 6 months after PTX using the radial‐arm water maze (Figure 4a) following our published protocol (Yabluchanskiy et al., 2020). In brief, we compared the learning performance of mice in each group by analyzing the day‐to‐day changes in the path length (Figure 4a,b). During acquisition, mice from all groups showed a decrease in the path length across days (without any significant change in velocity, Figure 4c), indicating learning of the task. We confirmed that learning was significantly impaired when tested 6 months after PTX (Figure 4b). To assess hippocampal encoded memory function at 6 months after PTX treatment, we also assessed the combined error rate, calculated by adding one error for each incorrect arm entry as well as for every 15 s spent not exploring the arms. The error rate for each mouse was then averaged among each experimental group in every trial block. Memory recall 7 days later was impaired in PTX treated mice, which also showed impaired extinction ability on day 11 (*n* = 10–15) (Figure 4d). The extinction blocks describe the ability of the mouse to forget and re‐learn the task with a different platform location. These findings confirm that both learning plasticity and memory are impaired after PTX treatment. These data are consistent with the progressive development of CICI. Restoration of microvascular functions after administration of senolytic treatments positively affected cognitive function in PTX treated mice. Figure 4b,d show that both GCV and ABT263 treatment improved learning and memory functions in PTX treated mice. Mice treated with GCV or ABT263 after PTX showed better memory recall on day 7. Both GCV and ABT263 treated mice exhibited improved extinction ability on day 11 and better relearning (Figure 4d). Previously we showed that cognitive performance in control mice was not significantly affected by GCV or ABT263 (Yabluchanskiy et al., 2020). Analyses of noncognitive parameters revealed no improvement in swimming speed or non‐exploratory behavior (cumulative time mice spent not actively looking for the platform, e.g., floating) in PTX treated mice that were given senolytics (data not shown). In the Y maze test, the control mice entered the novel arm more often than the PTX treated mice following the intertrial interval (Figure S7). Spatial working memory and novelty‐seeking behavior were rescued in mice treated with GCV or ABT263 after PTX (Figure S7). PTX treatment did not affect velocity, grip strength, rotarod performance and gait function (Figure S7). Together, the results suggest that removal of senescent cells after PTX improved performance in the radial‐arm water maze and Y maze, which results from enhanced hippocampal‐dependent spatial learning and memory and not from changes in motor or motivational processes.

## 4 DISCUSSION

The key findings of this study are that (1) PTX induces senescence in endothelial cells, which associates with microvascular rarefaction, NVC dysfunction and BBB disruption and that (2) both pharmacological (ABT263/Navitoclax) and genetic depletion of senescent cerebromicrovascular endothelial cells rescues PTX‐induced cerebromicrovascular dysfunction and attenuates CICI.

Our studies show that a PTX treatment induces senescence in CMVECs. The clinical relevance of our findings is supported by studies characterizing the effects of PTX on cultured peripheral endothelial cells in connection to the extensive use of PTX‐coated balloons and drug‐eluting stents for cardiovascular indications (including treatment of intermittent claudication) (Dake et al., 2016). These studies confirm that PTX induces senescence in endothelial cells and exert potent antiangiogenic activity (Ota et al., 2009; Wang et al., 2003). The pathophysiological consequences of PTX‐induced cerebromicrovascular endothelial senescence are likely multifaceted.

Here, we demonstrate that cognitive impairment in PTX treated mice is associated with significant decline in microvascular density both in the hippocampus and the cortex. These brain regions are involved in cognitive processes and are particularly vulnerable to ischemia. Previous studies demonstrate that decreased capillarization in the cortex and hippocampi in various pathophysiological conditions predicts cognitive dysfunction in the absence of or preceding neurodegeneration (Ungvari et al., 2018). PTX is known to inhibit endothelial proliferation and angiogenic processes both in tumors (Lau et al., 2004) and in the normal cardiovascular system (Wang et al., 2003). Impaired angiogenesis due to increased endothelial senescence (Ungvari et al., 2018) is a likely mechanism involved in cerebromicrovascular rarefaction induced by PTX treatment. It is generally considered that cerebromicrovascular rarefaction leads to a decline in cerebral blood flow, compromising metabolic support for neural signaling (Ungvari et al., 2018). Future studies are warranted to determine how increased PTX‐induced microvascular senescence impacts basal cerebral blood flow in the model used. Importantly, there is prima facie evidence that chemotherapy with PTX associates with decreased perfusion in the frontal and parietal lobes in breast cancer patients (Nudelman et al., 2014), but the role of microvascular rarefaction in this phenomenon remains to be determined. In line with the idea that senescent cells play a role in age‐related diseases, research has shown that transplanting a small number of autologous senescent cells into young mice can lead to several pathologies commonly associated with aging (Xu et al., 2017). Significantly, these experiments have demonstrated that there is a time delay between the accumulation of senescent cells and the onset of functional effects, which is similar to the delayed effects resulting from the senescent cell burden caused by chemotherapy. To further enhance future studies, it may prove beneficial to investigate potential sex differences in PTX‐induced endothelial senescence.

The brain has limited energy reserves and during periods of neuronal activation regional oxygen and glucose delivery has to be adjusted to the significantly increased metabolic demand through NVC (Tarantini et al., 2016). Another important function of the cerebromicrovascular endothelium is to mediate NVC responses via release of vasodilator NO in response to neuronal and astrocytic activation (Tarantini, Balasubramanian, et al., 2021). We provide critical evidence that PTX‐induced endothelial senescence impairs NO mediated NVC responses. A key role for senescent endothelial cells in impaired functional hyperemia is demonstrated by the findings that their removal rescues the NO mediated component of NVC responses in PTX treated mice. We posit that rescue of NVC responses contributes to the cognitive benefits conferred by senolytic treatments in the present study. Several lines of evidence support this concept. First, there are studies reporting that in breast cancer survivors PTX treatment induces generalized endothelial dysfunction (Watanabe et al., 2015) and impairs NVC responses, which predict cognitive decline (Carlson et al., 2018). Second, there is strong preclinical evidence demonstrating that in mice NVC responses positively correlate with cognitive performance (Febo & Foster, 2016) and that pharmacological inhibition of endothelium‐mediated NVC responses impairs cognition (Tarantini et al., 2015, 2017), mimicking CICI. Third, biological aging is also associated with increased presence of senescent endothelial cells in the cerebral microcirculation (Kiss et al., 2020, 2022) and their removal by senolytic treatments also confers neurovascular protection and cognitive benefits (Tarantini, Balasubramanian, et al., 2021).

Endothelial cells in the cerebral microvessels are an integral part of the BBB, where they exhibit extensive tight junctions to prevent leakage of blood borne factors into the brain parenchyma. Intact BBB function is required for normal structural and functional brain connectivity, synaptic activity, and information processing (Sweeney et al., 2019). Our studies provide evidence that PTX‐induced endothelial senescence leads to persistent BBB disruption associated with increased neuroinflammation, both of which can be reversed by senolytic treatments. Important data obtained in BubR1 hypomorphic (BubR1^(H/H)^) mice, which display senescence cell‐dependent phenotypes, provide additional evidence that cellular senescence is causally linked to BBB disruption (Yamazaki et al., 2016). Previous studies show that neuroinflammation associated with disruption of BBB integrity promotes neuronal/synaptic dysfunction and cognitive decline (Sweeney et al., 2019). Thus, we posit that improvement of BBB integrity also contributes to the cognitive benefits conferred by senolytic treatments.

CMVECs are connected via gap junctions and form a functional syncytium (Ungvari et al., 2018). They share cytoplasmic signals and thus one senescent endothelial cell can modulate both directly and indirectly (through paracrine factors) the function and phenotype of neighboring cells. Thus, increased presence of senescent CMVECs is expected to alter the function of a large portion of the microcirculatory network. Likewise, removal of senescent cells is expected to exert beneficial effects on the entire cerebral microcirculation. On the basis of our scRNA‐seq data other cell types may also be affected by PTX‐induced senescence, although apparently to a lesser extent. Further studies are needed to assess senescence‐related changes in these cells after PTX treatment and elucidate their role in the pathogenesis of CICI.

In conclusion, our findings show that senolytics confer neurovascular protection in PTX treated mice. Removal of senescent cells by treatment with navitoclax/ABT263 rescued NVC responses and BBB integrity and increased capillary density in brains of PTX treated mice, which likely contribute to improved cognitive performance. Our findings highlight the potential use of senolytic drugs as therapies to prevent CICI along with other side effects of chemotherapy (Yao et al., 2020). Our results have important translational relevance. Navitoclax/ABT263 (Shi et al., 2011) is in clinical trials as an orally active experimental anti‐cancer drug (Bertino et al., 2021; Budhraja et al., 2017; Rudin et al., 2012; Tan et al., 2011). Several clinical trials with various other senolytics, including BCL‐xL inhibitors, the flavonoid fisetin and the senolytic combination of dasatinib and quercetin, are underway. Thus, future clinical trials using senolytics are feasible and hold promise for preserving cerebrovascular health and cognitive function in chemotherapy patients. As senolytic therapy is a relatively new treatment, its long‐term effects are not yet known. Some possible side effects of senolytic therapy that have been speculated include altered immune responses and potential damage to healthy tissues. It is important to note that these potential side effects have not been widely observed in human clinical trials, and more research is needed to fully understand the safety and efficacy of senolytic therapy in humans. It is worth noting that the induction of endothelial senescence is linked with various stresses and conditions in humans, beyond chemotherapy. This fact provides a compelling reason to explore the potential effects of senolytics on the cerebral microcirculation in those conditions as well. Moreover, in situations where there is already an elevated burden of senescent cells at baseline, such as with advanced age, obesity, smoking, etc., the acquisition of even more senescent cells through chemotherapy may hasten the onset of cognitive decline. This acceleration may cause more noticeable clinical effects to manifest due to the threshold for cognitive decline being surpassed much earlier. In support of this concept, there is evidence to suggest that smoking (Wefel et al., 2004) and obesity (Janelsins et al., 2011) can exacerbate the cognitive effects of chemotherapy.

## AUTHOR CONTRIBUTIONS

The authors confirm contribution to the paper as follows: Study conception and design: CA, ANT, ST, AY, QT, PB, JC, ZU, and AC; data collection: CA, ANT, JD, RG, JF, ST, AKC, PB, AU, and FY; analysis and interpretation of results: CA, ANT, JD, RG, JF, ST, TK, AKC, PB, AU, RN, FY, QT, PM, TC, AY, JC, ZU, and AC; draft manuscript preparation: CA, ANT, JD, RG, JF, ST, TK, AKC, PB, AU, RN, FY, QT, PM, TC, AY, JC, ZU, and AC. All authors reviewed the results and approved the final version of the manuscript.

## CONFLICT OF INTEREST STATEMENT

None declared.

## Supporting information


Data S1

## Data Availability

Data Availability StatementThe data that support the findings of this study are available from the corresponding author upon reasonable request.
