# Supplementary material for: Accelerated cerebromicrovascular senescence contributes to cognitive decline in a mouse model of paclitaxel (Taxol)‐induced chemobrain
Source: Aging Cell. 2023 May 26;22(7):e13832. doi: 10.1111/acel.13832 (PMC10352561; doi:10.1111/acel.13832)
Supplement: Supplementary file 1 — Data S1 [file ACEL-22-e13832-s001.docx]

**SUPPLEMENTAL INFORMATION**

**SUPPLEMENTAL FIGURES**

**
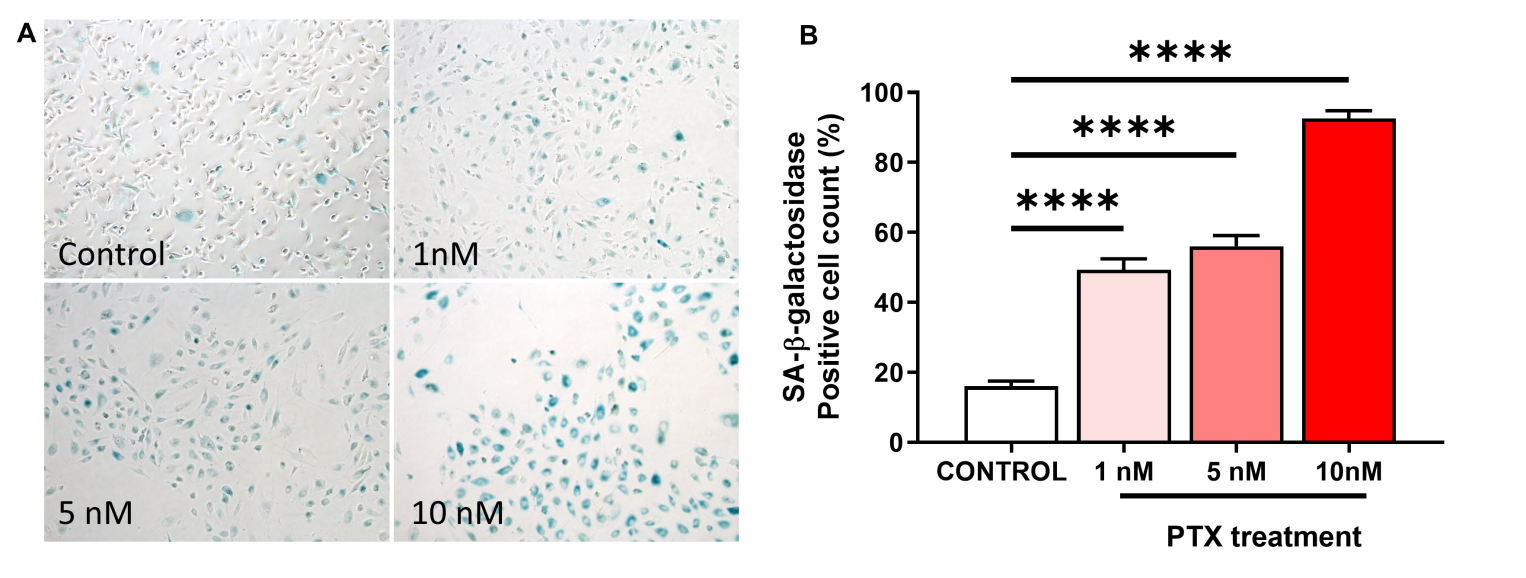
**

**Figure S1. PTX-induced senescence in cultured cerebromicrovascular endothelial cells.** (A) PTX induces cellular senescence in cultured CMVECs. CMVECs were treated with increasing concentrations of PTX and stained for senescence associated (SA)-β-galactosidase activity 7 days post treatment. (B) Bar graphs depict the percentage of SA-β-galactosidase positive (blue) CMVECs. ****P<0.0001 vs. non-treated controls. Data are mean±SEM (n=6 for each data point).

**
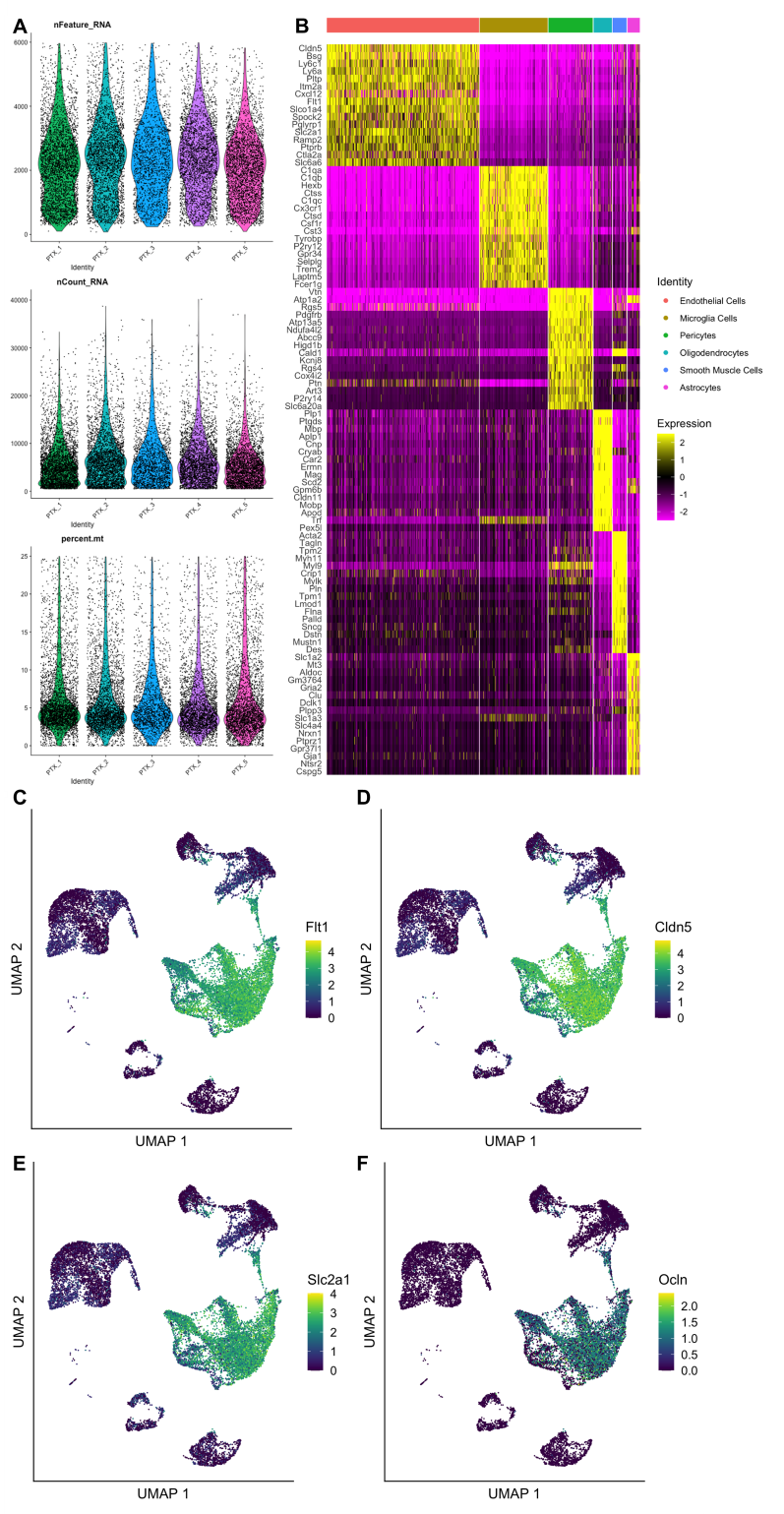
**

**Figure S2. (A)** Quality control measures (read count, feature count, ratio of mitochondrial genes) show that there is no sequencing bias between the individual samples. **(B)** The top 16 marker genes that are most highly expressed in the individual clusters. **(C-D)** Marker panel of canonical endothelial cell markers. Relative expression values for each cell in each cluster identified in the two-dimensional UMAP plots are shown. The canonical endothelial cell markers Flt1 (C), Cldn5 (D) and Slc2a1 (E) exhibit consistent labeling of cerebromicrovascular endothelial cells, while some other canonical endothelial cell markers (e.g. Ocln, (F)) exhibit poorer labeling of these cells using this methodology.

**
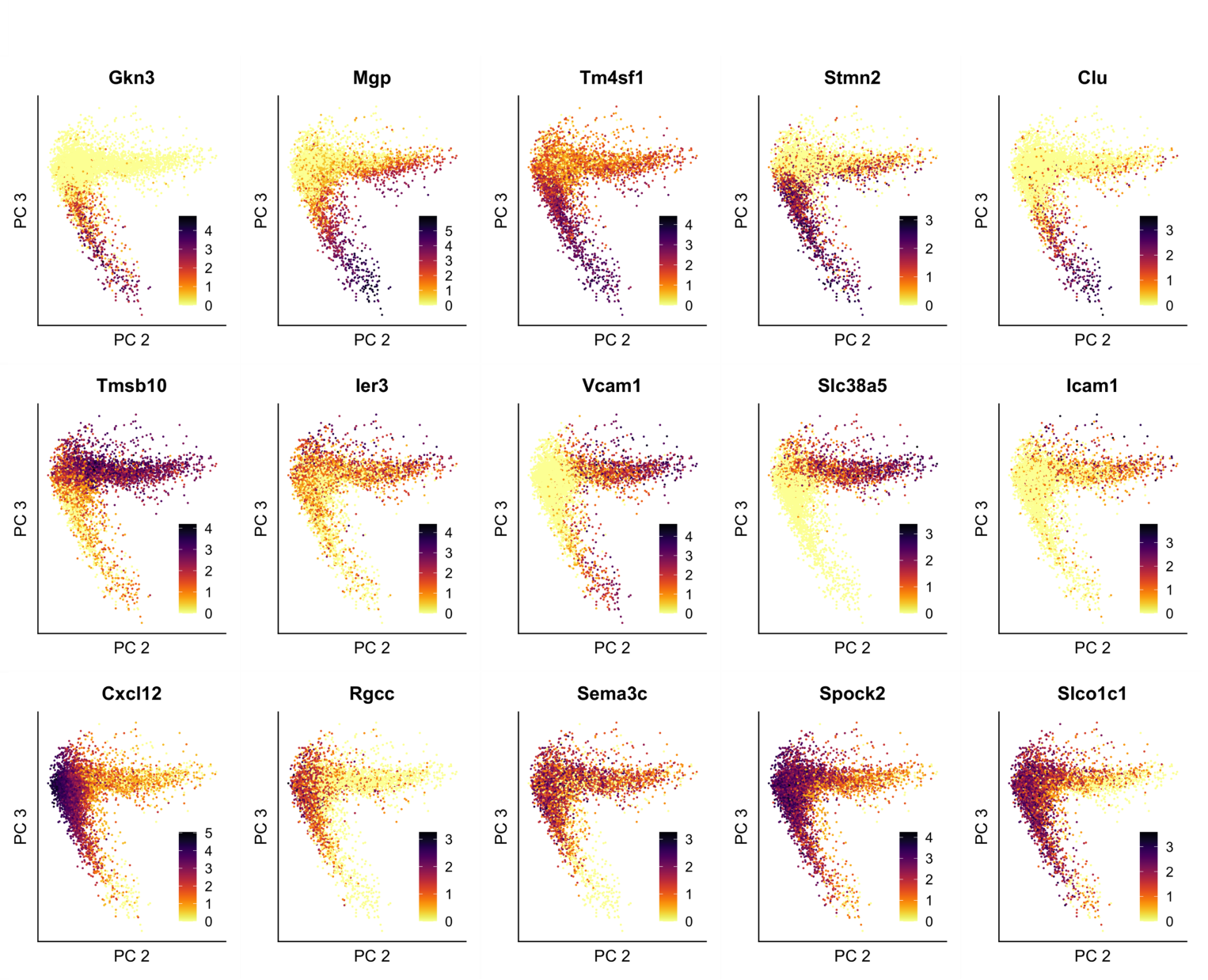
**

**Figure S3. Marker panel of endothelial cell subcluster-specific markers.** Principal component analysis of RNA-Seq data generated from brain endothelial cells. Shown is visualization of expression of marker genes of capillary-, venous- and arterial endothelial cell subclusters. Relative expression values for each cell in each cluster identified in the two-dimensional PCA plots are shown.


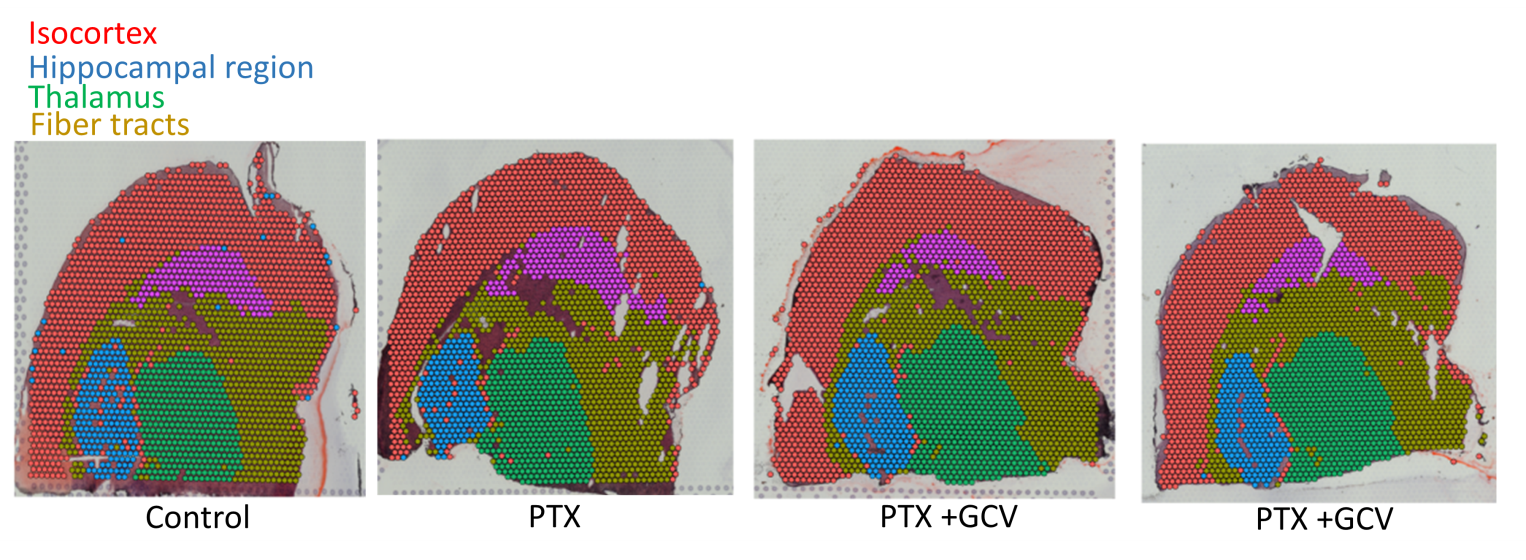


**Figure S4. Spatially-resolved clustering and gene expression in the brains of mice. A)** Maps of transcriptomic anatomy. Coronal sections of brains derived from control p16-3MR mice and PTX treated p16-3MR mice that received vehicle, ganciclovir (GCV) or ABT263 were H&E stained, imaged, then processed through the Visium Spatial Gene Expression workflow. Shown are image overlays containing data for spatially naïve clustering based on total differentially expressed genes. The discovered transcriptomic patterns correspond to neuroanatomically distinct regions.


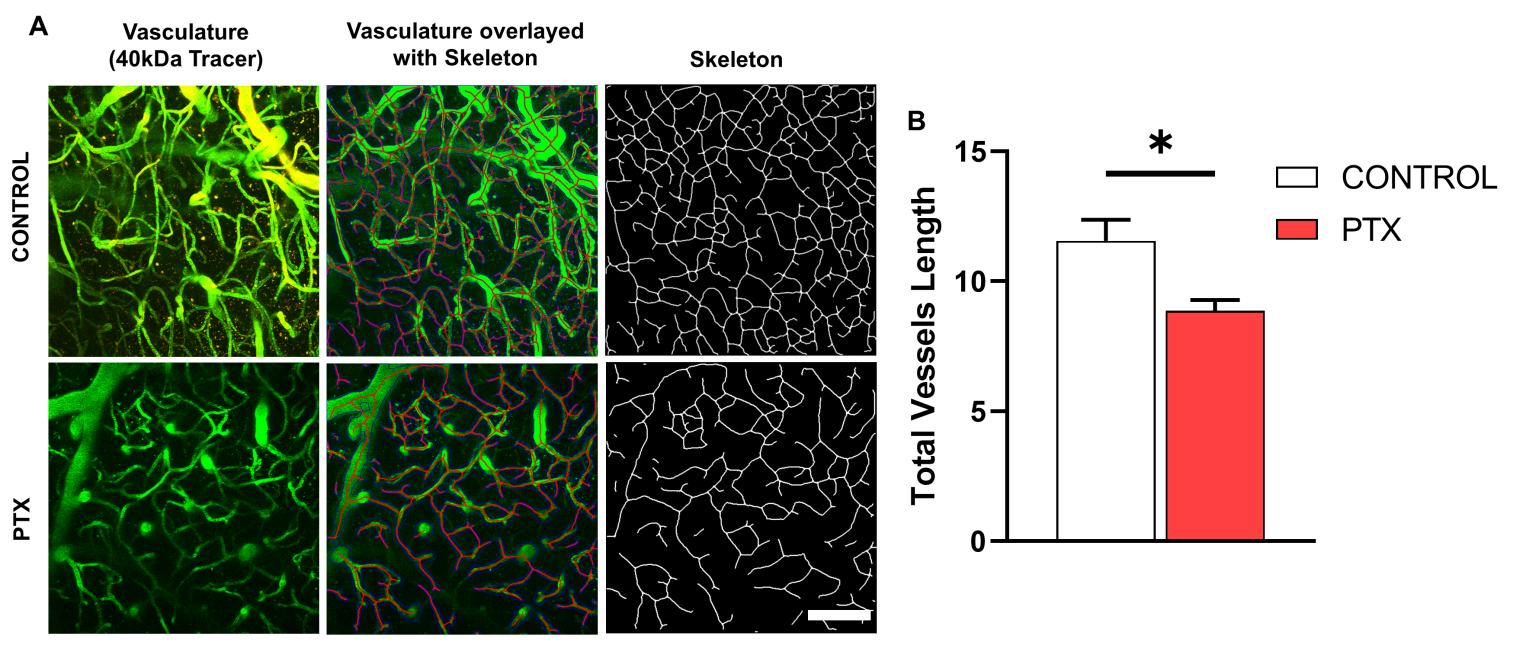


**Figure S5. Demonstration of PTX-induced cerebromicrovascular rarefaction by intravital two-photon microscopy. (A)** Segmentation of blood vessels in two-photon images. Original z-stack images captured in brains of control and PTX treated mice (red fluorescence: WGA-Alexa594 staining of the glycocalyx of the endothelial cells) were maximum projected and processed with a modified macro from a published ImageJ plugin^[1](#_ENREF_1)^. Skeleton images were used to calculate indices corresponding to microvascular density. Scale: 100 μm. (B)

**
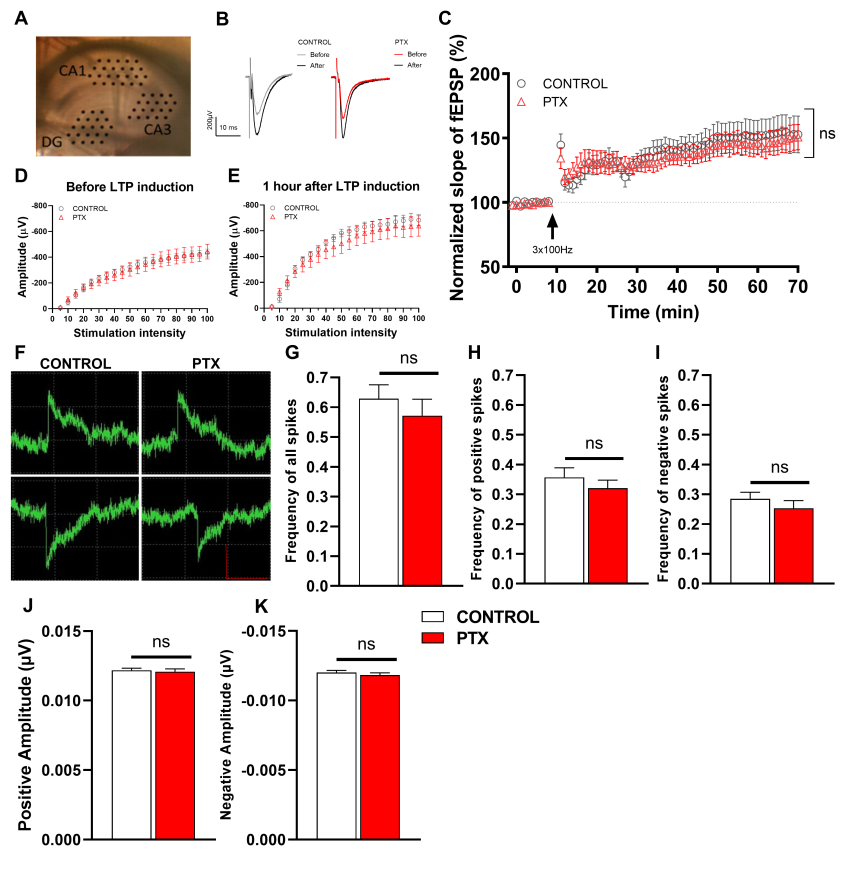
**

**Figure S6**: **PTX treatment does not affect neuronal function and synaptic activity.** PTX treatment does not cause any change in spontaneous activity and long term potentiation in hippocampal synapses. (A) Picture of hippocampal slice in MED 64 probe with electrodes positioned on the dentate gyrus (DG), CA3 and CA1 regions. (B) Synaptic potentiation of hippocampal CA1 synapses in PTX treated mice. Representative traces show EPSPs after (black line) and before (blue line for control, red line for treated) 100Hz stimulation to induce LTP in slices from control (left) and PTX treated (right) mice. Panel C: Normalized fEPSP showing LTP induced in both groups of mice. LTP was seen in both control mice (blue circles, n = 10 slices from 5 mice, 152.9 ± 13.4% last 10 min of recording), and PTX treated mice (red triangles, n = 9 slices from 5 mice, 147.3 ± 9.5% last 10 min of recording). Each data point represents the average of two successive test responses. The vertical arrow indicates the period of three 100 Hz stimulation. (Data shown as mean ± SEM. ns p > 0.05, Mann Whitney test). (D-E) Baseline and potentiated synaptic strength in PTX treated mice. Graph depicts amplitude of fEPSP evoked from control brain slices (blue circles) and brian slices from PTX treated mice (red triangles) by stepwise increase in the stimulus from 5 to 100 µA. Comparison of amplitudes between slices obtained from brains of control and PTX treated mice before LTP (upper panel, ns p > 0.05) and after LTP induction (lower panel, ns p > 0.05, Data shown as mean ± SEM). Both groups showed an increase in the fEPSP amplitudes after 1 hr of LTP induction. (E) Representative traces from spontaneous events in brain slices obtained from control (left panel) and PTX treated mice (right panel). There was no difference in the amplitudes of positive spikes (upper panel) and negative spikes (lower panel) between the two groups. Scale bars *y*=20 µV, *x*=2 ms. (F-H) No difference in the average frequency of all spikes in the hippocampi of control (n=7 slices from 5 mice) and PTX treated mice (n=8 slices from 5 mice). In addition, there was no difference in average frequency of positive spikes (middle panel) and average frequency of negative spikes (right panel, shown as mean ± SEM. ns p>0.05, Wilcoxon signed rank test). (I-J) Analysis of spontaneous event amplitudes (shown as mean ± SEM). There was no difference in the amplitudes of positive spikes (left panel, ns p>0.05, Wilcoxon signed rank test) and negative spikes (right panel, ns p>0.05, Wilcoxon signed rank test) between the two groups.

**
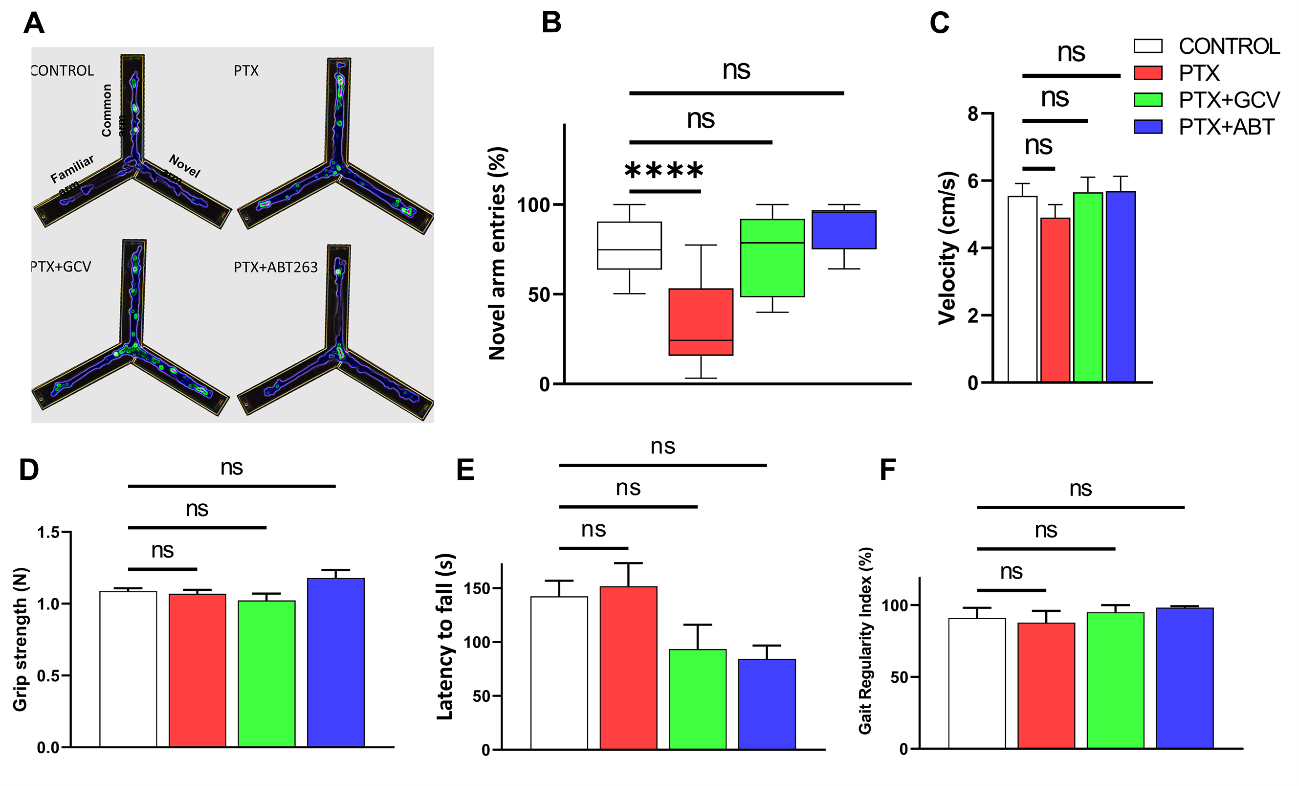
**

**Figure S7.** **Performance of PTX treated p16-3MR mice in behavioral tests.** Control p16-3MR mice and PTX treated p16-3MR mice that received vehicle, ganciclovir (GCV) or ABT263 were tested in the Y maze. (A) Schematic picture of the Y maze. The start arm (common arm), the familiar arm and the novel arm are indicated (see details in text). Representative search paths of a randomly selected animal from each group are shown (red). (B) Number of entries in novel arm of the Y-maze as percentage of all entries during the retrieval trial (n=8-39 for each datapoint; ****P<0.0001 vs. control). PTX treated mice exhibited significant impairment of contextual memory as indicated by a similar number of entries in the novel and previously encountered arms during retrieval in the delayed-alternation task. The performance of PTX treated animals that received GCV or ABT263 did ot differ significantly from that of control animals on this task. (C): Average velocity of mice was similar in each group. (D) PTX treatment in mice does not affect grip strength. (E) PTX teatment in mice does not effect performance on the rotarod test. (F) PTX teatment in mice does not affect gait performance. Shown are the regularity indices for each group. Data are mean±S.E.M.(n=8-39 for each datapoint). Statistical significance was calculated using one-way ANOVA with Tukey’s post hoc test to determine differences among groups.

**SUPPLEMENTAL TABLES**

**Supplemental Table S1:** List of cell-type specific markers[^2^](#_ENREF_2). EC: endothelial cells

| **Cell Type** | **ENTREZ ID** | **Symbol** | **Gene Name** |
| --- | --- | --- | --- |
| EC | 12741 | *Cldn5* | claudin 5 |
| EC | 14254 | *Flt1* | FMS-like tyrosine kinase 1 |
| EC | 28250 | *Slco1a4* | solute carrier organic anion transporter family, member 1a4 |
| Microglia | 13051 | *Cx3cr1* | chemokine (C-X3-C motif) receptor 1 |
| Microglia | 70839 | *P2ry12* | purinergic receptor P2Y, G-protein coupled 12 |
| Microglia | 231633 | *Tmem119* | transmembrane protein 119 |
| Pericytes | 18596 | *Pdgfrb* | platelet derived growth factor receptor, beta polypeptide |
| Pericytes | 16523 | *Kcnj8* | potassium inwardly-rectifying channel, subfamily J, member 8 |
| Oligodendrocytes | 18417 | *Cldn11* | claudin 11 |
| Oligodendrocytes | 12799 | *Cnp* | 2',3'-cyclic nucleotide 3' phosphodiesterase |
| smooth muscle cells | 11475 | *Acta2* | actin, alpha 2, smooth muscle, aorta |
| smooth muscle cells | 17880 | *Myh11* | myosin, heavy polypeptide 11, smooth muscle |
| Astrocytes | 11829 | *Aqp4* | aquaporin 4 |
| Astrocytes | 20511 | *Slc1a2* | solute carrier family 1 (glial high affinity glutamate transporter), member 2 |

**Supplemental Table S2:** List of senescence marker genes[^3^](#_ENREF_3)^,^ [^4^](#_ENREF_4)

| **Gene symbol** | **ENTREZ ID** | **Gene Name** |
| --- | --- | --- |
| *Cdkn2a* | 12578 | cyclin dependent kinase inhibitor 2A |
| *Bmi1* | 12151 | BMI1 proto-oncogene, polycomb ring finger |
| *Trp53* | 22059 | transformation related protein 53 |
| *Hmga1* | 15361 | high mobility group AT-hook 1 |
| *Chek1* | 12649 | checkpoint kinase 1 |
| *Chek2* | 50883 | checkpoint kinase 2 |
| *Prodh* | 19125 | proline dehydrogenase 1 |
| *Tnfrsf10b* | 21933 | TNF receptor superfamily member 10b |
| *Cdkn1a* | 12575 | cyclin dependent kinase inhibitor 1A |
| *Dao* | 13142 | D-amino acid oxidase |

**Supplemental Table S3:** List of endothelial cell (EC) sub-cluster specific genes[^5^](#_ENREF_5).

| **Endothelial cell sub-clasters** | **Gene symbol** | **ENTREZ ID** | **Gene Name** |
| --- | --- | --- | --- |
| arterial/arteriolar EC | *Gkn3* | 68888 | gastrokine 3 |
| arterial/arteriolar EC | *Stmn2* | 20257 | stathmin-like 2 |
| arterial/arteriolar EC | *Clu* | 12759 | clusterin |
| venous EC | *Icam1* | 15894 | intercellular adhesion molecule 1 |
| venous EC | *Slc38a5* | 209837 | solute carrier family 38, member 5 |
| venous EC | *Tmsb10* | 19240 | thymosin, beta 10 |
| capillary EC | *Cxcl12* | 20315 | chemokine (C-X-C motif) ligand 12 |
| capillary EC | *Rgcc* | 66214 | regulator of cell cycle |
| capillary EC | *Spock2* | 94214 | sparc/osteonectin, cwcv and kazal-like domains proteoglycan 2 |

**SUPPLEMENTAL MATERIALS AND METHODS**

*Experimental animals and experimental design*

To identify and eliminate senescent cells, we used a novel transgenic mouse model (p16-3MR mice[^6^](#_ENREF_6)^,^ [^7^](#_ENREF_7)) that carry a trimodal fusion protein (3MR) under the control of the p16*^Ink4a^* promoter. 3MR contains functional fragments of Renilla luciferase, which allows us to detect senescent cells in living animals, monomeric red fluorescent protein (mRFP), which enables us to FACS sort senescent cells from tissues, and the herpes simplex virus thymidine kinase, which allows us to selectively kill p16-positive senescent cells by administering the prodrug ganciclovir (GCV). Previous studies have extensively characterized this model[^8^](#_ENREF_8).

Three month old male p16-3MR mice were housed 3 per cage in the specific pathogen free animal facility at the University of Oklahoma Health Sciences Center (OUHSC). Animals were kept on a 12 hour light/dark cycle and fed standard rodent chow and water *ad libitum*, following standard husbandry techniques. One week before initiation of PTX treatment, mice were transferred to the conventional animal facility of the University of Oklahoma Health Sciences Center and housed under similar conditions.

Mice received PTX (5 mg/kg/day, i.p., n=150) or vehicle (DMSO+saline, n=50) in 2 cycles (5 days/cycle) with a 7-day interval between cycles. Mice were then left to recover for 2 weeks in the original environment. Then, PTX treated mice were assigned randomly to three groups. Two groups received the senolytic drug ABT263 (Navitoclax, Chemgood, C-1009, i.p., 1.5mg/kg/daily in DMSO/saline)[^6^](#_ENREF_6) or ganciclovir (GCV [TSZCHEM, RG001, >99%]; i.p. 25 mg/kg/daily) for 5 days and for 2 cycles with a 2 week interval between cycles[^6^](#_ENREF_6). The third group served as vehicle controls. To test for non-specific effects of senolytic treatments, a separate control cohort of p16-3MR mice animals received GCV or ABT263 without PTX pretreatment.

To detect toxic effects of the pharmacological treatments, changes in body weight and neurological status of the animals were monitored for 3 months post-treatment. Neurological examination was performed as reported ^[9](#_ENREF_9)^, by assessing each animal’s spontaneous activity, symmetry in the movement of the four limbs, forelimb outstretching, climbing ability, body proprioception, and response to vibrissae touch. Each examined animal was provided with a score calculated by the summation of all individual test scores (maximum18 points). Mice with a neurological score of 16 or less were excluded from the study.

At the end of the recovery period, 6 months after the PTX protocol, mice were tested for cognitive function and NVC responses or BBB integrity, then euthanized for tissue collection. All animal protocols were approved by the Institutional Animal Care and Use Committee of the OUHSC.

*Radial arms water maze testing*

To determine how senescence induced by PTX and depletion of senescent cells affect cognitive function, spatial memory and long term memory were tested by assessing performance in the radial arms water maze at 6 months post-chemobrain, following our published protocols[^6^](#_ENREF_6)^,^ [^10-12^](#_ENREF_10). The maze consists of 8 arms with a submerged escape platform at the end of one arm. Food coloring was added to the water to make it opaque. The maze was surrounded by privacy blinds with extramaze visual cues. Intramaze visual cues were placed at the end of the arms. Mice were monitored by video tracking directly above the maze and parameters were measured using Ethovision software (Noldus Information Technology Inc., Leesburg, VA, USA). Experimenters were blinded to the experimental conditions of the mice. Each day, mice were given the opportunity to learn the location of the submerged platform during 2 session blocks, each consisting of 4 consecutive acquisition trials. On each trial, the mouse was started in an arm not containing the platform and allowed to wade for up to 1 minute to find the escape platform. All mice spent 30 seconds on the platform following each trial before beginning the next trial. The platform was located in the same arm on each trial. Over 3 days of training, mice gradually improved performance. Upon entering an incorrect arm (all 4 paws within the distal half of the arm), the mouse was charged an error. Learning was assessed by comparing performance on days 2 and 3 of the learning period. When both groups learned the task, mice were placed in their home cage for 7 days. Then, they were given a recall trial on day 10. On day 11 (extinction), mice were tested for ability to relearn the task when the platform had been moved to a different arm not adjacent or diametrically positioned to the previous location. Mice were tested for 2 session blocks, the second, consisting of 4 trials, was used for comparison.

*Spatial memory testing of mice in Y-maze*

Hippocampal-dependent contextual memory was tested with the Y-maze two-trial delayed alternation task according to our published protocol[^13^](#_ENREF_13). In brief, a Y-maze apparatus made up of three enclosed Plexiglas arms (40 cm length x 9 cm width x 16 cm height) with extra-maze visual cues around the maze, was used to assess recognition memory. The test consisted of two trials separated by an intertrial interval (4 h). All mice were transported to the behavioral testing room in their home cages at least 1 h before testing. In the first training (acquisition) trial, mice were placed in the maze facing the end of a randomly chosen start arm and allowed to explore the maze for 5 min with one of the arms closed (novel arm). Mice were returned to their home cage until the second (retrieval) trial, run with all three arms of the maze open. The time spent in each arm was measured from video recordings by investigators blinded to age and treatment. Mice were required to enter an arm with all four paws in order for it to be counted as an entry. The time spent in the novel arm was calculated as a percentage of the total time spent exploring all three arms during the 2 min retrieval trial. The maze was cleaned with 70% ethanol between trials. Animals that spent < 20% of trial time engaged in exploration were removed from the study.

*Grip strength, rotarod, gait analysis*

As an additional control, we determined how the pharmacological treatments affect muscle function in mice. We used the grip strength test was used to measure the maximal muscle strength of forelimbs of the mice. Forelimb grip strength was assessed using a grip strength meter (Chatillon Ametek Force Measurement, Brooklyn, NY) based on the manufacturer’s recommendations. The strength measurements of each group of mice were obtained three times by the same investigator. The maximum grip strength values were used for subsequent analysis.

Motor coordination was assessed by using an automated four-lane rotarod tool (Columbus Instruments, Columbus, OH) as described previously. [^14^](#_ENREF_14)^,^ [^15^](#_ENREF_15) Motor skill learning was evaluated by analyzing day-to-day changes in performance on the accelerating rotarod test. Mice were pre-trained on rotarod by placing them on moving rotarod at 10 rotation per minutes (rpm) until they performed at this speed for 3 minutes. On the days of testing, mice were acclimated to the testing room for at least 15 min before stating new trial and throughout the day maintain in their home cages to avoid any possible stress. The test phase consisted of 3 trials (separated by 15-min inter-trial intervals) per day for 4 days. The testing apparatus was set to accelerate from 4 to 40 rpm in 300 s. One mouse was then placed on each lane and the rotarod was started with an initial rotation of 4 rpm. The rotational velocity was set to increase every 10 s and the latency to fall was recorded in seconds by an infra-red beam across the fall path along with the max rpm sustained by each mouse and final output was plotted as a summary of record on day 4.

To determine the impact of PTX treatment on gait coordination, we tested the experimental groups of mice using an automated computer assisted method (CatWalk; Noldus Information Technology Inc.). Using the CatWalk system, the detection of paw placement patterns during volunteer running on an illuminated glass walkway by a camera placed under the glass surface provides an automated analysis of gait function and the spatial and temporal aspects of interlimb coordination[^10^](#_ENREF_10)^,^ [^16^](#_ENREF_16). Briefly, animals were trained to cross the walkway and then, in a dark and silent room (< 20 lux of illumination), animals were tested in twenty consecutive runs (to obtain >200 steps per each animals). Data were averaged across ~20 runs in which the animal maintained a constant speed across the walkway. After manual identification and labeling of each footprint the variability of the data has been assessed using quartile dispersion. We adopted a common outlier definition, labeling points more than 1.5 interquartile ranges away from the sample median as extreme values. After variability analyses, spatial and temporal gait parameters were calculated. The regularity index (%) is a fractional measure of inter-paw coordination, which expresses the number of normal step sequence patterns relative to the total number of paw placements. The formula of regularity index is: (normal step sequence patterns) x 4/(total number of paw placements) x 100 (%). In healthy, fully coordinated animalls its value is close to 100%.

*Intravital two-photon microscopy*

To assess BBB permeability and cerebromicrovascular density, mice were equipped with a chronic cranial window and intravital two-photon microscopy-based and optical coherence tomography (OCT[^17^](#_ENREF_17)) based imaging methods were used as previously described[^1^](#_ENREF_1).

Chronic cranial window surgery

Animals were anesthetized with 2-3 % isoflurane (ISOTHESIA, Henry Schein Animal Health, OH, USA) gas via inhalation with Surgivet Classic T3 vaporizer (Smiths Medical, Minneapolis, MN, USA) with 1-2 L/min flow rate before and during the surgery. Eye blink, toe, and tail pinch reflexes were monitored to determine the depth of anesthesia. The experimental animal was placed on a heating pad to maintain core body temperature during the procedures. The head of the animal was fixed in an adaptor for the stereotaxic frame by ear and nose bars. Eye ointment was applied to both eyes to prevent ocular dehydration during anesthesia. Hair removal lotion was applied to the top of the head. After hair removal, the surface of the skin was disinfected with 70% ethanol. Then, the skin was removed from the top of the skull. 2 % lidocaine (Sigma-Aldrich, MO, USA) solution was dripped onto the periosteum. The periosteum was removed from the exposed area of the skull with a blade and the area was scraped gently to establish a clean surface. To dry the surface of the skull it was wiped with a cotton wool stick. After an area 3-4 mm in diameter had been chosen over the sensorimotor cortex, the skull was gently thinned using a pneumatic dental drill (Foredom, Blackstone Industries, Bethel, CT, USA). During this procedure, the surface was cooled by dripping of cold, sterile PBS. Debris was blown off with compressed air. When the bone was thin enough (indicated by its slight movement when it was gently pushed), craniotomy was performed under a drop of sterile PBS. The surface of the dura mater was carefully wiped down and any accidental bleeding – caused by the craniotomy – was stopped with a gelatin Hemosponge (Goodwill Lifesciences, India) immersed in sterile PBS. The cranial window was dried carefully to remove excess fluid from the area. A glass coverslip (diameter: 5 mm, Thomas Scientific, Swedesboro, NJ, USA), which was previously soaked in 70% ethanol, was rinsed in PBS and applied to the surface of the dura mater to completely cover the cranial window. The coverslip was fixed to the skull with liquid adhesive and after the superglue bonded, the cranial window was secured with Jet Set-4 dental acrylic resin (Lang Dental, Wheeling, IL, USA). The resin rim created a small pool around the cranial window. At the end of the surgery, the animals were treated with buprenorphine (1 ml/kg body weight, i.p.; Zoopharm, WY, USA). Enrofloxacin (5 mg/kg body weight, s.c.; Baytril, Bayer, Germany) was administered as a prophylactic antibiotic. The animals were closely monitored until the anesthesia wore off and they regained consciousness. Intravital imaging studies were conducted at least two to three weeks after surgery.

Intravital two-photon microscopy

Two-photon imaging was performed to assess microvascular density and BBB integrity, as previously described[^1^](#_ENREF_1). Intravital imaging was preformed using a FluoView 1000 MPE (Olympus, Tokyo, Japan) two-photon microscope coupled with a MaiTai HP DeepSee-OL 690 nm-1040 nm (Spectra-Physics, San Jose, CA, USA) laser and a XLPLN25XWMP 25× water immersion objective (1.05 numeric aperture; Olympus, Tokyo, Japan). An 800 nm laser line was used for excitation. The emitted light was collected by PMT detectors. Three channels with the following filter sets were used: 420-460 nm, 495-540 nm, 575-630 nm.

Mice were anesthetized with isoflurane (2-2.5 % for induction, 1.5-2 % for maintenance; with 1-2 L/min flow rate). The heads of the animals were fixed using a stereotaxic frame. To label the vascular glycocalyx, Wheat Germ Agglutinin, Alexa Fluor™ 594 Conjugate (4 ml/kg body weight of 1 mg/ml WGA-A594, Thermo Fisher Scientific, MA, USA) was injected retro-orbitally. Imaging of the cortex was performed through the cranial window. WGA-A594 binds to the glycocalyx in the cortical microvasculature, enabling the visualization of the microvascular network architecture and the accurate identification of the vessel wall boundaries. On the basis of the microvascular architecture cortical areas for subsequent BBB integrity studies were identified and imaged. First, meningeal vessels were detected and imaging depth of zero was set to them. Superior pial vessels were used as reference points for later examination. Cerebral microvessels were examined at ~0-200 µm depth. The same laser intensity (5%) and detector sensitivity were used regardless of the depth in the tissue to maintain the reproducibility and comparability with other animals. Images captured immediately after WGA-A594 injection served as a no-tracer background intensity internal control. Using two-photon microscopy, image stacks deep into the brain tissue were captured with limited photobleaching and tissue phototoxicity, as fluorescence occurs only within the plane of focus.

To assess BBB integrity and determine the severity of BBB disruption, first a fluorescent tracer dye with higher molecular weight (4 ml/kg body weight of 2 mg/ml FITC-dextran 500 kDa; Sigma-Aldrich, MO, USA) was injected retro-orbitally. Imaging of the pre-set area was performed to detect extravasation of the high molecular weight tracer dye, indicating severe BBB disruption. This high molecular weight tracer was only used for single microvessel measurement since detectable extravasation is minimal and is hardly detectable with image subtraction analysis.

To detect mild BBB disruption, fluorescent tracer dyes with lower molecular weight (4 ml/kg body weight of 2 mg/ml FITC-dextran 40 kDa, 3 kDa, Thermo Fisher Scientific, MA, USA; and sodium fluorescein, Sigma-Aldrich, MO, USA) were injected in order of descending molecular weight and imaging of the predetermined area was performed following the aforementioned protocol.

For image subtraction analyses, the volumes of interests (VOIs) were selected based on observable microvessels. 508 µm × 508 µm × 50-150 µm (x, y, z) VOI was imaged for z-stacks. The corresponding pixel numbers were 512 × 512 × 31; thus, the spatial resolution was approximately 1 µm × 1 µm × 5 µm in x, y, z directions (objective point spread function (PSF) <.1.5 μm)[^18^](#_ENREF_18)

Image analysis

The images were analyzed using FIJI ImageJ 1.53C version (National Institutes of Health, USA) with a custom-made macro, following the method of Nyúl-Tóth et al[^1^](#_ENREF_1). In brief, imported TIFF images were assembled into time-series of z-stacks then these were 3D corrected for the proper alignment. WGA-A594 stained vascular images were used as a segmented binary mask to subtract from 3D shift background corrected Z-stack maximum intensity tracer images to remove the areas corresponding to intravascular volumes. This approach enables the selective measurement of extravasated tracer intensities at each time point separately. Intensity of the intravascular volumes was also measured by inversion and subtraction of the vascularization-segmented images from the tracer channel. As a last step, intensity of images post-subtraction was measured. Integrated density values changes were used for the calculations of relative permeability.

Determination of relative and absolute permeability of cerebral microvessels within a volume of brain tissue

Measured total area integrated density values from extravascular tracer fluorescent intensity in z-stack maximum projections were normalized to the baseline intensity (WGA-A594 without the tracers) to avoid imaging error-based distortion. Intensities were represented as a function of elapsed time between the first (only WGA-A594) and further (WGA-A594 and tracer) recordings. The given function was analyzed by the calculation of the “area under curve” (AUC) which enabled the comparison of the extravasated tracer quantities, resulting in a relative permeability difference between subjects. Permeability was measured at every single time point (~15/tracer) with their own area fraction and normalized intensity. Then, the apparent solute permeability for different tracers was calculated from the median value of the relevant time points.

*Intravital optical coherence tomography (OCT)*

In the same mice used for two-photon imaging, the cerebral microcirculation was also imaged at least with a one-day difference, using an OCT system to assess microvascular density as previously described[^1^](#_ENREF_1).

Animals were anesthetized as described with isoflurane (2-2.5 % for induction, 1.5-2 % for maintenance) and their heads were fixed using a stereotaxic frame as described above.

A high-speed swept-source optical coherence tomography (SS-OCT) system (VEG220, Thorlabs) was used for in vivo imaging of mouse cerebral cortex. The system was equipped with a ~1310 nm broadband wavelength-swept laser with a spectral bandwidth of ~100 nm and a wavelength-swept frequency of ~200 KHz, providing a measured axial resolution ~14 μm in air (~10 μm in mouse brain tissue)[^19^](#_ENREF_19)^,^ [^20^](#_ENREF_20). The sensitivity of system was ~105 dB at the focal point measured using a mirror. The incident light power onto the surface of cerebral cortex was ~10 mW. In this system, 97% of the laser output power was divided to the sample and reference arms, and 3% of the output was distributed to a Mach-Zehnder interferometer (MZI) to trigger the data acquisition. The laser beam was scanned over the cranial window on the mouse head using a pair of galvanometer scanning mirrors (GSM, X by Y), yielding a three-dimensional (3D) volumetric data set (X by Y by Z). An objective with ~25 mm working distance (OCT-LK3, Thorlabs) was used in this system, providing a lateral resolution of ~13 μm in air.

In order to acquire images of blood vessels in mouse cerebral cortex, the scanning protocol was optimized for OCT-based optical microangiography (OMAG) algorithm as previously described[^1^](#_ENREF_1). To acquire in vivo volumetric angiography in mouse cerebral cortex, the OMAG algorithm was employed for the acquisition of vascular information from the 3D OCT data set by coherently analyzing the intrinsic scattering property of moving red blood cells (RBCs) in blood vessels[^1^](#_ENREF_1). In the fast-scanning axis (X), 400 A-scans covering a length of ~2.5 mm constituted a B-scan frame. In the slow-scanning axis (Y), 400 sampling positions covering a length of ~2.5 mm contributed to a 3D data set (sampling resolution is 6.25 × 6.25 × 6.25 μm3 in X × Y × Z). In the scanning protocol, eight repeated B-scan frames at each sampling position were obtained to constitute a complete 3D scanning data cube with 960 by 400 by 3200 (Z by X by Y) voxels. The eight repeated B-scan frames were further utilized to generate a cross-sectional vascular map at each sampling position[^1^](#_ENREF_1)^,^ [^19^](#_ENREF_19)^,^ [^20^](#_ENREF_20). An enface summed intensity projection (SIP) algorithm [^21^](#_ENREF_21) allowing visualization of small decorrelation signals that overlaid major vessels was applied to the OMAG volume dataset to present the vessel network.

The image processing and calculation were processed by Python Programming Language (*Python Software Foundation,* <https://www.python.org/>) and ImageJ Fiji (*National Institutes of Health, Bethesda, Maryland, USA,* <https://imagej.nih.gov/ij/>). First, a two-dimensional (2D) *en face* raw OCTA image was generated through the SIP algorithm projected from the volumetric data of blood vessels produced by OMAG. Second, the input raw OCTA image was processed by the fast Fourier transform (FFT) to remove the stripe noise caused by the breath and heartbeat of mouse. Despeckle algorithm was used to eliminate the speckle noise. The adaptive threshold was applied in different regions of the entire raw OCTA image with different threshold values to adaptively filter noises in subareas. Third, Median Local Threshold was used on the filtered OCTA image to generate a binary OCTA map. Next, the edges of vessels were detected and contoured as the OCTA perimeter map. Last, a skeletonized OCTA map was generated by iteratively removing the pixels in the outer boundary of the binarized OCTA map until one pixel remained along the width direction of the vessels [^22^](#_ENREF_22). The quantification and analysis of blood vessels were performed through the quantitative parameters of vessel area density (VAD) and vessel skeleton density (VSD).

VAD was calculated by a unit-less ratio of the total image area occupied by the blood vessels to the total image area in the binarized OCTA maps.

$$VAD=\frac{\sum_{i=1, j=1}^{n} A_{(i,j)}}{\sum_{i=1,j=1}^{n} X_{(i,j)}}$$

Where, $A_{(i,j)}$ represented the pixel of binarized vessel area (white pixels on Fig. 3C), and $X_{(i,j)}$ represents all the pixels in the binarized OCTA image, and $(i,j)$ are the coordinates of pixels in the quantitative OCTA images ($i$ ~ row, $j$ ~ column). With this calculation, VAD provided an accurate estimate of real vessel density as it included both vessel length and diameter information.

Similarly, VSD was defined as the unit-less ratio of the total image area of the vessel length to the total image area in the skeletonized OCTA maps.

$$VSD=\frac{\sum_{i=1, j=1}^{n} S_{(i,j)}}{\sum_{i=1,j=1}^{n} X_{(i,j)}}$$

Where, $S_{(i,j)}$ represented the pixel of skeletonized vessel area, and $X_{(i,j)}$ represented all the pixels in the skeletonized OCTA image. VSD was an assessment of the vessel length density regardless of the vessel diameter.

*Assessment of neurovascular coupling responses*

NVC responses were assessed as described previously[^7^](#_ENREF_7)^,^ [^11^](#_ENREF_11). In brief, mice in each group were anesthetized with isoflurane (2% induction and 1% maintenance), endotracheally intubated and ventilated (MousVent G500; Kent Scientific Co, Torrington, CT). A thermostatic heating pad (Kent Scientific Co, Torrington, CT) was used to maintain rectal temperature at 37^o^C [^23^](#_ENREF_23). End-tidal CO_2_ was controlled between 3.2% and 3.7% to keep blood gas values within the physiological range, as described[^24^](#_ENREF_24)^,^ [^25^](#_ENREF_25). The right femoral artery was cannulated for arterial blood pressure measurement (Living Systems Instrumentations, Burlington, VT) [^23^](#_ENREF_23). Blood pressure was within the physiological range throughout the experiments (90-110 mmHg). Mice were immobilized and placed on a stereotaxic frame (Leica Microsystems, Buffalo Grove, IL), the scalp and periosteum were pulled aside and the skull was gently thinned using a dental drill while cooled with dripping buffer. A laser speckle contrast imager (Perimed, Järfälla, Sweden) was placed 10 cm above the thinned skull. To achieve the highest CBF response the right whiskers were stimulated for 30 seconds at 5 Hz from side to side. Differential perfusion maps of the brain surface were captured. Changes in CBF were assessed above the left barrel cortex in six trials in each group, separated by 5 min intervals. To assess the role of NO mediation, CBF responses to whisker stimulation were repeated after administrating the nitric oxide synthase inhibitor N^ω^-Nitro-L-arginine methyl ester (L-NAME). Changes in CBF were averaged and expressed as percent (%) increase from the baseline value. Experiments lasted <1 hour/mouse, which permitted stable physiological parameters to be obtained. In each study the experimenter was blinded to the treatment of the animals. At the end of the experiments the animals were transcardially perfused with ice-cold PBS and decapitated. The brains were immediately removed and samples were collected for subsequent studies. Whole brains were collected for FACS analysis and transcriptomics. Acute brain slices were collected for LTP measurements. Half brains were immersion fixed in 4% paraformaldehyde for 24 hours, transferred to sucrose gradients and embedded and cut for immunohistochemistry.

*Electrophysiological studies to assess synaptic function and long-term potentiation (LTP)*

To determine how PTX affects neuronal function, extracellular recordings were performed from acute hippocampal slices obtained from a separate cohort of control and PTX-treated animals as described[^16^](#_ENREF_16)^,^ [^26-28^](#_ENREF_26).

Briefly, horizontal hippocampal slices of 325 µm thickness from mice in each cohort were prepared in ice cold solution containing (in mmol/L) sucrose 110, NaCl 60, KCl 3, NaH_2_PO_4_ 1.25, NaHCO_3_ 28, sodium ascorbate acid 0.6, glucose 5, MgCl_2_ 7, CaCl_2_ 0.5 using a HM650V vibrating microtome (Thermo Scientific). Slices were then transferred to a holding chamber (Scientific Designs, Inc.) which contained oxygenated artificial cerebrospinal fluid (aCSF) of the following composition (in mmol/L) NaCl 126, KCl 2.5, NaH_2_PO_4_ 1.25, MgCl_2_ 2, CaCl_2_ 2, NaHCO_3_ 26, glucose 10, pyruvic acid 2, ascorbic acid 0.4. Slices were left to recover for at least 60 min at room temperature prior to recording in a brain slice chamber (Automate Scientific Inc., CA).

For recording network activity and extracellular field potentials, the slice was transferred to and positioned on a P5002A multi-electrode array system (Alpha MED Scientific Inc., Osaka, Japan) and perfused with aCSF at a rate of 2 ml/min, equilibrated with 95% O_2_ and 5% CO_2_ at 32°C. To secure contact between the slice and electrodes and to improve mechanical stability, a piece of nylon mesh and a slice anchor harp were placed on top of the slice. The slice was maneuvered to position the hippocampus on the array. After the slice had settled in the recording chamber, before applying any stimulation, six hundred (600) traces of network activity were recorded, each for a 1 sec duration, under physiological conditions with continuous aCSF perfusion[^29^](#_ENREF_29). Network activity data were analyzed as previously described[^30^](#_ENREF_30). Briefly, raw MED64 Mobius workflow files were opened and spikes were extracted in Mobius (WitWerx Inc.). Positive and negative spike-threshold was set to + 0.01 and − 0.01 mV, respectively. Spike traces were extracted along with 1 ms of baseline before and after the spike event, without down sampling. Raw data were filtered using a Bessel high-pass (2-pole) with a cutoff frequency of 1000 Hz and a DC filter with a typical spike length set to 1 ms. The resulting file containing all extracted spikes within that slice recording was then processed in Microsoft Excel using a visual basic macro that was coded and validated by us to extract each of the final parameters for statistical comparison.

Field excitatory postsynaptic potentials (fEPSPs) were generated in the CA1 region of the hippocampus by stimulating downstream electrodes in the CA1 and CA3 regions of the hippocampus along the Schaffer collateral pathway. Input/output curves (I/O curves) were generated by applying increasing stimulus currents to the pathway from 0 to 100µA and recording the responses. The threshold stimulus for generating fEPSPs was determined as 30%–40% of the stimulus strength needed to generate the maximum fEPSP amplitude during the I/O curve measurement. The slice was stimulated once every 30 seconds until a stable baseline lasting at least 10 minutes was observed. Long term potentiation (LTP) was induced using 100 high-frequency stimulation pulses at 100 Hz applied three times with 30-second intervals. Next, baseline stimulation was resumed and recorded fEPSPs for at least 60 more minutes. Finally, another I/O curve was recorded and generated as described above. For all recordings, we used the MED-64 system and Mobius software (Alpha MED Scientific Inc). Potentiation was calculated as the percent increase of the mean fEPSP descending slope (10–90 section) after high-frequency stimulation and normalized to the mean fEPSP descending slope of baseline recordings during 3 minutes prior tetanus.

*Determination of senescent cell burden by flow cytometric analysis*

We used sorted cells obtained from the single-cell suspensions from the brain samples to analyze senescent cell burden. Animals were sacrificed and transcardially perfused with the ice-cold PBS for 12 minutes. The brains were collected and transferred to ice cold PBS. Under the hood, the brain tissue was gently diced and digested in the mix of collagenase (Sigma), dispase I (Sigma, D4818), hyaluronidase (Sigma, H4272), and elastase (Sigma, E0127) in PBS for 30 minutes at 37°C with gentle and continuous agitation. Further, brain tissue was mechanically dissociated and run through 100 and 30 μm nylon mesh (Miltenyi Biotech) to prepare single-cell suspensions. Tissue debris and myelin were removed by the density gradient reagent 'Debris Removal Solution' (Miltenyi Biotech Inc. USA). Cells were fixed in 1% PFA (Santa Cruz Biotech), washed once with ice-cold PBS, resuspended in MACS buffer (Miltenyi Biotech) and stored at 4°C.

On the day of flow cytometry, fixed cells were stained with the RFP-Booster (AlexaFluor-488, 1:1000; Chromotek; US-QUO201590, 0.5gm/L) for 30 minutes, centrifuged (300×g, 10 min), and resuspended in MACS buffer (Miltenyi Biotech). The RFP-Booster allows for the detection of senescent cells that express RFP-containing 3MR construct under the control of the p16*^Ink4a^* promoter.

To assess senescent cell burden, a portion of the RFP-Booster-stained samples were also labelled with an antibody directed against the endothelium-specific surface marker CD31 (anti-CD31; clone: MEC13.3; 1:50; DyLight 594; NOVUSBIO NB600-1475DL594). First, the fraction of RFP+ senescent cells were determined as a percentage of total cells in the single cell suspensions from whole brain lysates using a Guava® EasyCyte™ BGR HT Flow Cytometer (Luminex). Then, the ratio of RFP^+^/CD31^+^ senescent endothelial cells as a percentage of all CD31^+^ endothelial cells was determined.

Fluorescent Activated Cell Sorting (FACS) with the low-pressure WOLF Cell Sorter™ (NanoCellect) was used to obtain the cell suspension enriched in brain senescent cells. Prior to cell sorting, the analysis of unstained cells was performed to gate RFP+ cells. RFP+ cells were sorted at low speed (100-400 events/s) to avoid cellular damage and provide a high sorting yield. Sorted cells were centrifuged (300 g, 10 minutes), supernatants were discarded, and cell pellets were resuspended in MACS buffer. Suspensions of sorted cells were kept in the dark at 4°C for further analysis. RFP+ senescent cells were stained with cell-specific markers. Antibodies directed against CD31 were used to quantify the ratio of senescent endothelial cells (anti-CD31; clone: MEC13.3; 1:50; DyLight 594; NOVUSBIO NB600-1475DL594). 130 μL of sorted cells were plated on a 96-well plate and incubated with 10 μL of 1% BSA (Sigma) in 2.8% triton X-100 surfactant (EMD Chemicals Inc., TX1568-1) for 15 minutes. The cellular profile of brain senescent cells was assessed on a Guava® EasyCyte™ BGR HT Flow Cytometer (Luminex). Data were analyzed using FCS Express software (De Novo Software). Ratio of senescent RFP+/CD31+ endothelial cells was determined as a percentage of total RFP+ senescent cells. Cell debris was gated out during sorting and flow cytometry data analysis.

*Assessing PTX-induced senescence in cultured cerebromicrovascular endothelial cells*

Cellular senescence is characterized by expression of senescence-associated β-galactosidase (SA-β-gal) activity. To assess the sensitivity of cultured primary cerebromicrovascular endothelial cells to PTX-induced senescence, we measured SA-β-gal activity in PTX-treated cultured human cerebromicrovascular endothelial cells (CMVECs). Human CMVECs were purchased from Cell systems and cultured in Complete Classic medium supplemented with Culture boost and BAC-off antibiotics (Cell systems). The cells were treated with vehicle (DMSO in media) or PTX (from 1nM to 25nM in media, diluted from 1mg/ml stock in DMSO) for 10 days. The media was replaced with fresh media containing vehicle or PTX once every 2 days during the treatment period. After the end of the treatment period, the cells were washed once with PBS and then fixed for SA-beta gal assay.

To assess the sensitivity of CMVECs to PTX-induced senescence, SA-β-gal activity was compared in PTX-treated CMVECs and untreated controls. On day 10 after PTX treatment, histochemical staining for SA-β-gal activity was performed using the Sigma-Senescence Cells Histochemical Staining Kit (Sigma, No. CS0030, St. Louis, MO), following the manufacturer’s guidelines. To analyze the ratio of senescent cells in each well, microscopic images of the stained CMVEC cultures were captured (at 10x magnification, 30 random fields per group). The percentage of β-galactosidase-positive cells (blue cytoplasmic staining) was calculated by a naïve observer.

*Single-cell transcriptomics*

To identify senescent cells in brains of PTX-treated mice, a single-cell transcriptomics-based method was also used, as described[^2^](#_ENREF_2). This technology enables capture of mRNAs from single cells obtained from dissociated tissues, synthesis and amplification of cDNA, and generation of single-cell libraries for sequencing. We used a gel bead-in-emulsion-based droplet sequencing method, which is ideal for studying a large amount of brain cells in an unbiased manner. We identified cerebromicrovascular endothelial cells and other brain cell types on the basis of their gene expression profile and matched transcriptomic signatures of cellular senescence to these cells, as described[^2^](#_ENREF_2).

Tissue processing, cell isolation

Brains from PTX-treated mice (n=5) were quickly removed and rinsed in ice cold PBS, and minced into ≈ 1 mm^3^ pieces. Single-cell suspensions were obtained from the brain samples using the methods described for the FACS studies, with some modifications. In brief, brain samples were digested and cleared with Debris Removal Solution (Miltenyi Biotech). Cell pellets were stained with SYTOX™ Green Nucleic Acid Stain (Invitrogen), and dead cells were removed by Fluorescent Activated Cell Sorting (FACS) with the low-pressure WOLF Cell Sorter™ (NanoCellect) to obtain the cell suspension enriched in living cells. Cells were kept on ice until the sequencing. The advantage of this method is that it yields intact, high quality cells that are ideal for transcriptomic studies.

Single-cell RNA sequencing (scRNA-seq)

All the samples were simultaneously isolated and processed through all steps to generate stable cDNA libraries. After dissociation, cells were diluted in ice-cold PBS containing 0.4% BSA. Cells were loaded into a Chromium Single Cell 3′ Chip (10x Genomics, Pleasanton, California) and processed following the manufacturer’s instructions. Library construction was performed using the Chromium Single Cell 3′ Library & Gel Bead Kit v2 (Catalog# 120267, 10x Genomics, Pleasanton, California). Libraries were pooled based on their molar concentrations. Pooled library was sequenced on one high-output lane of the NovaSeq 6000 instrument (Illumina, San Diego, California). To de-multiplex samples, process barcodes, align and filter reads and generate feature-barcode matrices we used 10x Genomics Cell Ranger (v3.0.2) pipeline (10x Genomics, Pleasanton, California) according to the manufacturer`s instructions. Reads were mapped to the 10x Genomics reference of mm10 mouse transcriptome (v.1.2.0).

Analysis of single-cell datasets:

The downstream analyses of Cell Ranger output were performed with the help of Seurat (v4.1) workflow implemented as an R package (R v4.1.1)[^31^](#_ENREF_31)^,^ [^32^](#_ENREF_32). Data obtained in each PTX-treated mouse samples were pooled. Our initial dataset contained 17728 cells. In the first step, low quality cells were removed. Cells with extremely high or low number of unique genes and cells with extremely high percentage of reads that map to the mitochondrial genome (more than 15% of all reads) were excluded from the further analysis[^33^](#_ENREF_33)^,^ [^34^](#_ENREF_34). After this quality control step, the final dataset consisted of data from 13487 cells. To remove technical variability, we normalized our data using *SCTransform* algorithm[^35^](#_ENREF_35) implemented natively in the Seurat workflow and boosted by the *glmGamPoi* method[^36^](#_ENREF_36). During this step the variable ‘percentage of reads that map to the mitochondrial genome’ were regressed out and the other parameters of the function were set to default. The top 3000 variable features detected by the *SCTransform* algorithm were used to run principal component analysis (PCA) on the data by the RunPCA function with the default parameters. The cells were clustered with by the *FindNeighbors* and the *FindClusters* function subsequently using the top 25 component of PCA. This function used unbiased Louvain clustering algorithm with the resolution parameter 0.065 (arXiv:0803.0476 [physics.soc-ph] accessed at https://arxiv.org/abs/0803.0476)[^37^](#_ENREF_37).

Clusters representing less than 3% of all cells were excluded from the downstream analysis. Cell clusters were identified by the expression of known, previously validated canonical cell type markers[^2^](#_ENREF_2) (Supplemental Table S1). Majority of cells were endothelial cells and microglia. Other cells types (pericytes, oligodendrocytes, vascular smooth muscle cells, astrocytes) were also represented in lower ratios. Visualization of pre-processed data was performed by uniform manifold approximation and projection algorithm (UMAP) implemented in the R package *uwot* (v0.1.4) and called by Seurat (v4.1) workflow (arXiv:1802.03426 [stat.ML] accessed at <https://arxiv.org/abs/1802.03426>).

Assessment of the expression of senescence-related genes

Our goal was to identify senescent cells on the basis of their gene expression profile. To achieve that goal, senescence-related gene expression was characterized at the individual cell level by calculating a modified enrichment score[^38^](#_ENREF_38) for each cell using the AUCell algorithm. The AUCell algorithm used a set of senescence-related core genes, compiled on the basis of the literature (Supplemental Table S2)[^2-4^](#_ENREF_2) as we previously described[^2^](#_ENREF_2). In brief, using this algorithm in each cells genes are ranked by their expression value and an "Area Under the Curve" (AUC) calculation is performed to determine whether a critical subset of the input gene set is enriched within the expressed genes for each cell[^39^](#_ENREF_39). As previously described, threshold was set based on the distributions of cell-specific senescence enrichment scores and a binary senescence variable (senescent or non-senescent) was defined for each cell.

For further analysis, endothelial cells were subset, PCA embedded, and sub-clustered within the same Seurat (v4.1) workflow. For the PCA embedding the RunPCA function was used with the default parameters. For the sub-clustering the top 20 PCA dimensions were selected and the resolution parameters were set to 0.2 in the subsequent FindNeighbors and FindClusters functions. 5 sub-clusters were identified which were subsequently categorized to 3 categories: endothelial cells form arteries (2 clusters), veins (2 clusters) and capillaries (1 cluster) using canonical marker genes (Supplemental Table S3)[^5^](#_ENREF_5).

*Spatial Transcriptomics*

A spatial transcriptomics (ST) -based method to assess spatial distribution of cellular senescence in brains of PTX-treated mice, as previously described[^40^](#_ENREF_40). Spatially resolved whole transcriptome mRNA expression was analyzed in sections of brains, while capturing histological information in the same tissue section. Microdomains containing senescent cells were identified on the basis of their senescence-related gene expression profiles and were mapped to different anatomical brain regions, including the isocortex, white matter and hippocampi as described[^40^](#_ENREF_40).

Tissue processing and RNA sequencing,

Brain tissue was harvested from mice (killed with CO_2_) that had been exsanguinated by transcardial PBS perfusion[^12^](#_ENREF_12). The brains were quickly removed and rinsed in ice cold PBS. Tissues were then cryoprotected in 30% sucrose diluted in 1X PBS, embedded in OCT and stored at −80°C. Cryosections were cut at 10μm thickness onto Visium Spatial Gene Expression Slides (10x Genomics), with micro-printed, densely packed, uniquely barcoded, RNA-capture oligomer grids. Following an initial H&E stain and imaging, tissues were permeabilized to capture RNA on the grid array. Quantitative cDNA libraries were prepared from permeabilized tissue sections placed on the Capture Areas of a Visium Spatial Gene Expression Slides using the manufacturer's protocol, then sequenced using NovaSeq S4 platform (NovaSeq 6000) using the XP chemistry. Each spatial voxel, or ~55μm “spot” on the grid array, is uniquely barcoded for spatial mapping of transcripts to the initial H&E image. Spots are 100 μm apart, center-to center. To limit technical variation, all the brain slices were processed and sequenced at the same time, using identical conditions.

Read alignment, annotation, and quantification

The proprietary Space Ranger software (10x Genomics) was used to perform the read alignment, annotation, and estimation of read counts from FASTQ files and demultiplexing. Gene expression was estimated per spot, and fiducial alignment of voxels to physical location on microscope slide images was performed utilizing anatomical annotation region (AAR) tags (Space Ranger) and microscope slide images, generating a spatially-mapped feature-barcode matrix for each sample.

Analysis of ST datasets

A feature-barcode matrix of all voxels for each of the eight samples was generated in which each feature indicates a specific transcript; each barcode represents a spatial voxel. Among all the capture spots of each capture area of the Visium slides, roughly two thirds of them were both present on brain tissue and had detectable gene expression. After matrix generation, all downstream analyses were performed with the Seurat v4.0 package[^31^](#_ENREF_31)^,^ [^41^](#_ENREF_41). The dataset from each sample was normalized individually with the SCTransform^[35](#_ENREF_35)^ function implemented in the Seurat package, using the default parameters, then integrated into a single dataset. All pre-processing steps were performed on the integrated dataset after quality control steps to remove spots with low read counts, low feature counts (less than 200), and high ratios of mitochondrial genes (more than 30%). After an initial linear dimension reduction step performed using the Seurat RunPCA function, clustering was performed using the top 20 principal components and the resolution factor 0.15 using the default graph-based community detection algorithm implemented in the form of the FindNeighbor and FindClusters functions in the Seurat package[^42^](#_ENREF_42). The annotation of the expression clusters was performed anatomically using the H&E stained histological images and the Allen Brain Atlas.

Spots with high levels of senescence were identified based on the expression of *Cdkn2a*. Differential gene expression between the spot of interest and the other spots were calculated using the MAST (Model-based Analysis of Single Cell Transcriptomics) method implemented as a R/Bioconductor package MAST[^43^](#_ENREF_43).

*Detection of activated microglia by immunohistochemistry*

Brains were perfusion-fixed (4% ice-cold paraformaldehyde; at 100 mmHg). Frozen OCT-embedded sagittal sections (35 μm) were cut and stored free-floating in cryoprotectant solution (25% glycerol, 25% ethylene glycol, 25% of 0.1 m phosphate buffer, and 25% water) at −20 °C. Sections were rinsed with Tris-buffered saline (TBS), permeabilized with TBS with 0.05% Tween-20. Antigen retrieval was achieved using 10 mM citrate buffer (10 mm sodium citrate and 0.05% Tween 20, pH 6.0) at 90 °C for 20 min followed by 3x washes with TBST. After blocking with 5% BSA and 1% fish gelatin in TBS at room temperature for 2 h, sections were immunolabeled for IBA-1 (rabbit anti-mouse Iba-I antibody; 1:200, Fijifilm; overnight at 4°C) and the endothelial marker endomucin (rat monoclonal anti-mouse endomucin antibody; 1:50, Invitrogen; overnight at 4°C) to identify microglia and capillary endothelial cells in the brain, respectively. Sections were washed for 5 minutes (3x) with TBST followed by incubation with goat anti-rabbit IgG (Invitrogen; Alexa 532), goat anti-rat IgG (Invitrogen; Alexa 488) for 2 h at room temperature. The sections were then washed with TBST for 5 minutes (3X), followed by nuclear staining with DAPI (5 mg/ml, Invitrogen, 1:10,000) for 5 minutes. Finally, the sections were washed and mounted on to slides using Prolong antifade mounting medium. Confocal images were obtained using Leica SP8 MP confocal laser scanning microscope. The relative staining intesity for IBA-1 positive perivascular microglia per region of interest in the cortex and hippocampi was assessed. In each animal 4 randomly selected fields were analyzed in 6 nonadjacent sections.

*Statistical analysis*

Depending on the experiment, statistical analyses were carried out by unpaired t test, one-way or two-way ANOVA with Fisher LSD post hoc test using GraphPad Prism 7.0, as appropriate. Differences were considered significant at p < 0.05. Data are presented as means ± standard error of mean (SEM) or all measured values as bar graphs or box plots with interquartile distributions and median values. Analysis was made with GraphPad Prism.

**SUPPLEMENTAL REFERENCES**

1. Nyul-Toth A, Tarantini S, DelFavero J, Yan F, Balasubramanian P, Yabluchanskiy A, Ahire C, Kiss T, Csipo T, Lipecz A, Farkas AE, Wilhelm I, Krizbai IA, Tang Q, Csiszar A and Ungvari Z. Demonstration of age-related blood-brain barrier disruption and cerebromicrovascular rarefaction in mice by longitudinal intravital two-photon microscopy and optical coherence tomography. *Am J Physiol Heart Circ Physiol*. 2021;320:H1370-H1392.

2. Kiss T, Nyul-Toth A, Balasubramanian P, Tarantini S, Ahire C, DelFavero J, Yabluchanskiy A, Csipo T, Farkas E, Wiley G, Garman L, Csiszar A and Ungvari Z. Single-cell RNA sequencing identifies senescent cerebromicrovascular endothelial cells in the aged mouse brain. *Geroscience*. 2020;42:429-444.

3. Carnero A. Markers of cellular senescence. *Methods Mol Biol*. 2013;965:63-81.

4. Nagano T, Nakano M, Nakashima A, Onishi K, Yamao S, Enari M, Kikkawa U and Kamada S. Identification of cellular senescence-specific genes by comparative transcriptomics. *Sci Rep*. 2016;6:31758.

5. Kalucka J, de Rooij L, Goveia J, Rohlenova K, Dumas SJ, Meta E, Conchinha NV, Taverna F, Teuwen LA, Veys K, Garcia-Caballero M, Khan S, Geldhof V, Sokol L, Chen R, Treps L, Borri M, de Zeeuw P, Dubois C, Karakach TK, Falkenberg KD, Parys M, Yin X, Vinckier S, Du Y, Fenton RA, Schoonjans L, Dewerchin M, Eelen G, Thienpont B, Lin L, Bolund L, Li X, Luo Y and Carmeliet P. Single-Cell Transcriptome Atlas of Murine Endothelial Cells. *Cell*. 2020;180:764-779 e20.

6. Yabluchanskiy A, Tarantini S, Balasubramanian P, Kiss T, Csipo T, Fulop GA, Lipecz A, Ahire C, DelFavero J, Nyul-Toth A, Sonntag WE, Schwartzman ML, Campisi J, Csiszar A and Ungvari Z. Pharmacological or genetic depletion of senescent astrocytes prevents whole brain irradiation-induced impairment of neurovascular coupling responses protecting cognitive function in mice. *Geroscience*. 2020;42:409-428.

7. Tarantini S, Balasubramanian P, Delfavero J, Csipo T, Yabluchanskiy A, Kiss T, Nyul-Toth A, Mukli P, Toth P, Ahire C, Ungvari A, Benyo Z, Csiszar A and Ungvari Z. Treatment with the BCL-2/BCL-xL inhibitor senolytic drug ABT263/Navitoclax improves functional hyperemia in aged mice. *Geroscience*. 2021;43:2427-2440.

8. Demaria M, O'Leary MN, Chang J, Shao L, Liu S, Alimirah F, Koenig K, Le C, Mitin N, Deal AM, Alston S, Academia EC, Kilmarx S, Valdovinos A, Wang B, de Bruin A, Kennedy BK, Melov S, Zhou D, Sharpless NE, Muss H and Campisi J. Cellular Senescence Promotes Adverse Effects of Chemotherapy and Cancer Relapse. *Cancer Discov*. 2017;7:165-176.

9. Fulop GA, Ahire C, Csipo T, Tarantini S, Kiss T, Balasubramanian P, Yabluchanskiy A, Farkas E, Toth A, Nyúl-Tóth Á, Toth P, Csiszar A and Ungvari Z. Cerebral venous congestion promotes blood-brain barrier disruption and neuroinflammation, impairing cognitive function in mice. *Geroscience*. 2019;41:575-589.

10. Ungvari Z, Tarantini S, Hertelendy P, Valcarcel-Ares MN, Fulop GA, Logan S, Kiss T, Farkas E, Csiszar A and Yabluchanskiy A. Cerebromicrovascular dysfunction predicts cognitive decline and gait abnormalities in a mouse model of whole brain irradiation-induced accelerated brain senescence. *Geroscience*. 2017;39:33-42.

11. Tarantini S, Valcarcel-Ares NM, Yabluchanskiy A, Fulop GA, Hertelendy P, Gautam T, Farkas E, Perz A, Rabinovitch PS, Sonntag WE, Csiszar A and Ungvari Z. Treatment with the mitochondrial-targeted antioxidant peptide SS-31 rescues neurovascular coupling responses and cerebrovascular endothelial function and improves cognition in aged mice. *Aging Cell*. 2018;17.

12. Tarantini S, Valcarcel-Ares MN, Toth P, Yabluchanskiy A, Tucsek Z, Kiss T, Hertelendy P, Kinter M, Ballabh P, Sule Z, Farkas E, Baur JA, Sinclair DA, Csiszar A and Ungvari Z. Nicotinamide mononucleotide (NMN) supplementation rescues cerebromicrovascular endothelial function and neurovascular coupling responses and improves cognitive function in aged mice. *Redox Biol*. 2019;24:101192.

13. Csiszar A, Tucsek Z, Toth P, Sosnowska D, Gautam T, Koller A, Deak F, Sonntag WE and Ungvari Z. Synergistic effects of hypertension and aging on cognitive function and hippocampal expression of genes involved in beta-amyloid generation and Alzheimer's disease. *Am J Physiol Heart Circ Physiol*. 2013;305:H1120-30.

14. Tarantini S, Valcarcel-Ares NM, Yabluchanskiy A, Fulop GA, Hertelendy P, Gautam T, Farkas E, Perz A, Rabinovitch PS, Sonntag WE, Csiszar A and Ungvari Z. Treatment with the mitochondrial-targeted antioxidant peptide SS-31 rescues neurovascular coupling responses and cerebrovascular endothelial function and improves cognition in aged mice. *Aging cell*. 2018;17:e12731.

15. Yabluchanskiy A, Tarantini S, Balasubramanian P, Kiss T, Csipo T, Fülöp GA, Lipecz A, Ahire C, DelFavero J, Nyul-Toth A, Sonntag WE, Schwartzman ML, Campisi J, Csiszar A and Ungvari Z. Pharmacological or genetic depletion of senescent astrocytes prevents whole brain irradiation-induced impairment of neurovascular coupling responses protecting cognitive function in mice. *Geroscience*. 2020;42:409-428.

16. Tarantini S, Hertelendy P, Tucsek Z, Valcarcel-Ares MN, Smith N, Menyhart A, Farkas E, Hodges EL, Towner R, Deak F, Sonntag WE, Csiszar A, Ungvari Z and Toth P. Pharmacologically-induced neurovascular uncoupling is associated with cognitive impairment in mice. *J Cereb Blood Flow Metab*. 2015;35:1871-81.

17. Li YD, Choi WJ, Wei W, Song SZ, Zhang QQ, Liu JL and Wang RK. Aging-associated changes in cerebral vasculature and blood flow as determined by quantitative optical coherence tomography angiography. *Neurobiol Aging*. 2018;70:148-159.

18. Negrean A and Mansvelder HD. Optimal lens design and use in laser-scanning microscopy. *Biomed Opt Express*. 2014;5:1588-609.

19. Tang Q, Wang J, Frank A, Lin J, Li Z, Chen CW, Jin L, Wu T, Greenwald BD, Mashimo H and Chen Y. Depth-resolved imaging of colon tumor using optical coherence tomography and fluorescence laminar optical tomography. *Biomed Opt Express*. 2016;7:5218-5232.

20. Tang Q, Nagaya T, Liu Y, Horng H, Lin J, Sato K, Kobayashi H and Chen Y. 3D mesoscopic fluorescence tomography for imaging micro-distribution of antibody-photon absorber conjugates during near infrared photoimmunotherapy in vivo. *J Control Release*. 2018;279:171-180.

21. Allegrini D, Montesano G, Fogagnolo P, Pece A, Riva R, Romano MR and Rossetti L. The volume of peripapillary vessels within the retinal nerve fibre layer: an optical coherence tomography angiography study of normal subjects. *Br J Ophthalmol*. 2018;102:611-621.

22. Su L, Ji Y, Tong NT, Sarraf D, He XG, Sun XD, Xu X and Sadda S. Quantitative assessment of the retinal microvasculature and choriocapillaris in myopic patients using swept-source optical coherence tomography angiography. *Invest Ophth Vis Sci*. 2020;61.

23. Toth P, Tarantini S, Tucsek Z, Ashpole NM, Sosnowska D, Gautam T, Ballabh P, Koller A, Sonntag WE, Csiszar A and Ungvari ZI. Resveratrol treatment rescues neurovascular coupling in aged mice:role of improved cerebromicrovascular endothelial function and down-regulation of NADPH oxidas. *Am J Physiol Heart Circ Physiol*. 2014;306:H299-308.

24. Tarantini S, Hertelendy P, Tucsek Z, Valcarcel-Ares MN, Smith N, Menyhart A, Farkas E, Hodges E, Towner R, Deak F, Sonntag WE, Csiszar A, Ungvari Z and Toth P. Pharmacologically-induced neurovascular uncoupling is associated with cognitive impairment in mice. *J Cereb Blood Flow Metab*. 2015;35:1871-81.

25. Toth P, Tarantini S, Ashpole NM, Tucsek Z, Milne GL, Valcarcel-Ares NM, Menyhart A, Farkas E, Sonntag WE, Csiszar A and Ungvari Z. IGF-1 deficiency impairs neurovascular coupling in mice: implications for cerebromicrovascular aging. *Aging Cell*. 2015;14:1034-44.

26. Oka H, Shimono K, Ogawa R, Sugihara H and Taketani M. A new planar multielectrode array for extracellular recording: application to hippocampal acute slice. *J Neurosci Methods*. 1999;93:61-7.

27. Liu CC, Tsai CW, Deak F, Rogers J, Penuliar M, Sung YM, Maher JN, Fu Y, Li X, Xu H, Estus S, Hoe HS, Fryer JD, Kanekiyo T and Bu G. Deficiency in LRP6-mediated Wnt signaling contributes to synaptic abnormalities and amyloid pathology in Alzheimer's disease. *Neuron*. 2014;84:63-77.

28. Tucsek Z, Noa Valcarcel-Ares M, Tarantini S, Yabluchanskiy A, Fulop G, Gautam T, Orock A, Csiszar A, Deak F and Ungvari Z. Hypertension-induced synapse loss and impairment in synaptic plasticity in the mouse hippocampus mimics the aging phenotype: implications for the pathogenesis of vascular cognitive impairment. *Geroscience*. 2017.

29. Nagaraja RY, Sherry DM, Fessler JL, Stiles MA, Li F, Multani K, Orock A, Ahmad M, Brush RS, Anderson RE, Agbaga MP and Deak F. W246G Mutant ELOVL4 Impairs Synaptic Plasticity in Parallel and Climbing Fibers and Causes Motor Defects in a Rat Model of SCA34. *Mol Neurobiol*. 2021;58:4921-4943.

30. Hopiavuori BR, Deak F, Wilkerson JL, Brush RS, Rocha-Hopiavuori NA, Hopiavuori AR, Ozan KG, Sullivan MT, Wren JD, Georgescu C, Szweda L, Awasthi V, Towner R, Sherry DM, Anderson RE and Agbaga MP. Homozygous Expression of Mutant ELOVL4 Leads to Seizures and Death in a Novel Animal Model of Very Long-Chain Fatty Acid Deficiency. *Mol Neurobiol*. 2018;55:1795-1813.

31. Stuart T, Butler A, Hoffman P, Hafemeister C, Papalexi E, Mauck WM, 3rd, Hao Y, Stoeckius M, Smibert P and Satija R. Comprehensive Integration of Single-Cell Data. *Cell*. 2019;177:1888-1902 e21.

32. Butler A, Hoffman P, Smibert P, Papalexi E and Satija R. Integrating single-cell transcriptomic data across different conditions, technologies, and species. *Nat Biotechnol*. 2018;36:411-420.

33. Ilicic T, Kim JK, Kolodziejczyk AA, Bagger FO, McCarthy DJ, Marioni JC and Teichmann SA. Classification of low quality cells from single-cell RNA-seq data. *Genome Biol*. 2016;17:29.

34. Luecken MD and Theis FJ. Current best practices in single-cell RNA-seq analysis: a tutorial. *Mol Syst Biol*. 2019;15:e8746.

35. Hafemeister C and Satija R. Normalization and variance stabilization of single-cell RNA-seq data using regularized negative binomial regression. *Genome Biol*. 2019;20:296.

36. Ahlmann-Eltze C and Huber W. glmGamPoi: fitting Gamma-Poisson generalized linear models on single cell count data. *Bioinformatics*. 2021;36:5701-5702.

37. Blondel VD, Guillaume JL, Lambiotte R and Lefebvre E. Fast unfolding of communities in large networks. *J Stat Mech-Theory E*. 2008.

38. Subramanian A, Tamayo P, Mootha VK, Mukherjee S, Ebert BL, Gillette MA, Paulovich A, Pomeroy SL, Golub TR, Lander ES and Mesirov JP. Gene set enrichment analysis: a knowledge-based approach for interpreting genome-wide expression profiles. *Proc Natl Acad Sci U S A*. 2005;102:15545-50.

39. Van de Sande B, Flerin C, Davie K, De Waegeneer M, Hulselmans G, Aibar S, Seurinck R, Saelens W, Cannoodt R, Rouchon Q, Verbeiren T, De Maeyer D, Reumers J, Saeys Y and Aerts S. A scalable SCENIC workflow for single-cell gene regulatory network analysis. *Nat Protoc*. 2020;15:2247-2276.

40. Kiss T, Nyul-Toth A, DelFavero J, Balasubramanian P, Tarantini S, Faakye J, Gulej R, Ahire C, Ungvari A, Yabluchanskiy A, Wiley G, Garman L, Ungvari Z and Csiszar A. Spatial transcriptomic analysis reveals inflammatory foci defined by senescent cells in the white matter, hippocampi and cortical grey matter in the aged mouse brain. *Geroscience*. 2022.

41. Hao Y, Hao S, Andersen-Nissen E, Mauck WM, 3rd, Zheng S, Butler A, Lee MJ, Wilk AJ, Darby C, Zager M, Hoffman P, Stoeckius M, Papalexi E, Mimitou EP, Jain J, Srivastava A, Stuart T, Fleming LM, Yeung B, Rogers AJ, McElrath JM, Blish CA, Gottardo R, Smibert P and Satija R. Integrated analysis of multimodal single-cell data. *Cell*. 2021;184:3573-3587 e29.

42. Waltman L and van Eck NJ. A smart local moving algorithm for large-scale modularity-based community detection. *Eur Phys J B*. 2013;86.

43. Finak G, McDavid A, Yajima M, Deng J, Gersuk V, Shalek AK, Slichter CK, Miller HW, McElrath MJ, Prlic M, Linsley PS and Gottardo R. MAST: a flexible statistical framework for assessing transcriptional changes and characterizing heterogeneity in single-cell RNA sequencing data. *Genome Biol*. 2015;16:278.
